# Clinical temporal relation extraction with long-context transformers: a robustness study on MIMIC-III and MIMIC-IV

**DOI:** 10.3389/fdgth.2026.1878461

**Published:** 2026-08-26

**Authors:** Swetha V. Padmavathi Polisetty, Deepthi Godavarthi

**Affiliations:** School of Computer Science and Engineering, VIT-AP University, Amaravati, Andhra Pradesh, India

**Keywords:** clinical natural language processing, electronic health records, inter-dataset evaluation, long-context transformers, temporal relation extraction

## Abstract

**Introduction:**

Temporal relation extraction from electronic health records is important for patient timeline construction and longitudinal clinical analysis, yet remains challenging because temporally relevant evidence may be distributed across extended clinical narratives.

**Methods:**

This study investigated whether long-context clinical transformers offer practical advantages for rule-based event-pair temporal relation classification across matched and transfer-oriented evaluation settings. Event-pair datasets were constructed from MIMIC-III and MIMIC-IV discharge summaries, with temporal labels assigned using a deterministic rule-based pipeline. The task was formulated as a four-class classification problem using BEFORE, AFTER, OVERLAP, and NONE labels. Clinical-Longformer and Clinical-BigBird were evaluated as primary long-context models, while BERT-base, BioBERT, Bio_ClinicalBERT and BiomedBERT served as standard-context baselines. To address GPU memory constraints during 4,096-token fine-tuning, a progressive two-stage strategy was used, in which long-context models were first fine-tuned at 1,024 tokens and then continued at 4,096 tokens.

**Results:**

Experiments covered in-domain, zero-shot inter-dataset, and adaptation-based transfer settings. Clinical-BigBird achieved the best MIMIC-III in-domain macro-F1 of 93.85% and the strongest performance in both adaptation settings. Clinical-Longformer achieved the best MIMIC-IV in-domain macro-F1 of 97.32% and the strongest zero-shot MIMIC-IV-to-MIMIC-III macro-F1 of 92.36%. BERT-base and BioBERT reproduced the same directional transfer pattern, with stronger MIMIC-III-to-MIMIC-IV zero-shot results and improvements after adaptation. The 4,096-token continuation stage consistently outperformed 1,024-token training alone. Physician review confirmed 160 of 200 rule-derived labels as correct, corresponding to 80.0% agreement. Statistical testing supported selected improvements, although not all pairwise differences were significant.

**Conclusion:**

Long-context modeling was particularly useful for improving robustness to dataset shift and target-domain adaptation in clinical temporal relation extraction. However, the findings should be interpreted in the context of rule-derived silver labels.

## 1 Introduction

Electronic health records (EHRs) have become an essential resource for data-driven clinical research because they preserve large volumes of patient information in both structured and unstructured formats. In particular, narrative clinical documents, such as discharge summaries, progress notes, radiology reports, and longitudinal care records, contain rich information on symptoms, diagnoses, procedures, medications, laboratory findings, and treatment outcomes. However, much of this clinically meaningful evidence remains embedded in unstructured free text, which limits its direct computational use. Therefore, clinical natural language processing (NLP) methods are needed to transform narrative documentation into structured and analyzable knowledge (1, 2). Temporal information extraction is important in this setting because clinical interpretation depends not only on identifying events, but also on determining when they occurred and how they are related over time (3, 4).

Temporal relation extraction plays a central role in clinical NLP because meaningful interpretation of patient records requires chronological reasoning over symptoms, diagnoses, procedures, medications, and outcomes. Accurate temporal relation identification can support patient timeline construction, treatment history analysis, disease progression modeling, and other downstream applications that rely on coherent longitudinal interpretation of clinical narratives (5, 6). Nevertheless, clinical temporal relation extraction remains difficult because temporally relevant evidence is often implicit, distributed across multiple sentences, and expressed through specialized medical language. In many cases, the temporal relation between two clinical events depends on contextual cues that may occur outside the immediate event mentions, making purely local sentence-level modeling insufficient for reliable classification (7, 8).

This challenge becomes more pronounced when prepared event-pair contexts are lengthy enough to exceed the input limit of standard transformer encoders. Standard BERT-style biomedical models are often limited to 512 tokens, potentially truncating relevant temporal evidence before inference is complete. Such context loss is problematic in EHR narratives because the information required to determine whether one event occurred before, after, or at the same time as another may be distributed across broader sections of the surrounding clinical context. To address this limitation, recent work has explored long-sequence pretrained clinical transformers, including Clinical-Longformer and Clinical-BigBird, as well as broader long-clinical-text modeling approaches that emphasize the value of preserving extended contextual dependencies (9, 10). In parallel, prompt-based temporal relation extraction and large-language-model-based clinical information extraction studies have expanded the methodological landscape; however, recent findings also show that robust inference over complex clinical narratives remains challenging (11, 12).

Recent shared-task research on chemotherapy treatment timeline extraction has further highlighted the practical significance of temporal relation modeling in realistic longitudinal oncology narratives and has shown that temporal extraction is a clinically meaningful component of larger patient-level timeline construction pipelines (13). The 2025 ChemoTimelines shared task extended this direction by building upon the initial 2024 challenge and further examining temporal relation extraction, timeline construction, and supervised fine-tuning strategies for clinical narratives (14). Recent work has also investigated large-language-model fine-tuning for temporal relation extraction from clinical notes, indicating growing interest in adapting generative models for domain-specific temporal reasoning tasks (15).

Despite these advances, important gaps remain. MIMIC-III and MIMIC-IV are major publicly available critical-care EHR resources containing deidentified clinical data and free-text notes, making them useful corpora for evaluating temporal relation extraction across related but distinct EHR datasets (1, 16). However, to the best of our knowledge, prior work has not systematically compared long-context clinical transformers with standard-context baselines representing general-domain, biomedical, and clinical pretraining for rule-derived event-pair temporal relation classification across both MIMIC-III and MIMIC-IV under in-domain, zero-shot inter-dataset, and adaptation-based transfer settings. Existing studies have contributed substantially to clinical temporal relation extraction, long clinical sequence modeling, and timeline extraction, but focused empirical evidence is still needed to determine whether preserving extended event-pair context improves temporal relation classification and robustness across EHR datasets.

To address this gap, this study presents a robustness-oriented evaluation framework for rule-derived clinical temporal relation extraction using existing long-context clinical and standard-context pretrained transformer architectures. The task is formulated as a four-class event-pair classification problem using the labels BEFORE, AFTER, OVERLAP, and NONE. In this study, NONE indicates event pairs for which the rule-based timestamp-resolution pipeline could not assign a deterministic temporal relation. The study compares the long-context Clinical-Longformer and Clinical-BigBird models with four standard 512-token baselines: BERT-base, BioBERT, Bio_ClinicalBERT, and BiomedBERT. The evaluation is conducted across MIMIC-III and MIMIC-IV using in-domain testing, zero-shot inter-dataset transfer, and adaptation-based transfer to assess both predictive performance and robustness under dataset variation.

The novelty of this study is methodological and empirical rather than architectural. The study does not introduce a new transformer architecture. Instead, it provides a unified comparison of long-context clinical transformers and standard-context pretrained transformers across MIMIC-III and MIMIC-IV using a four-class silver-label formulation, subject-disjoint data partitions, in-domain evaluation, bidirectional zero-shot transfer, and adaptation-based transfer under a common experimental protocol. In contrast, previous studies have primarily focused on manually annotated benchmark datasets, three-class relation formulations, single-dataset evaluation, timeline-generation tasks, graph-based models, or prompt-based approaches.

### 1.1 Scope of this research

This research focuses on rule-based extraction of clinical temporal relations from prepared event-pair instances derived from large-scale EHR corpora. The task is formulated as a silver-label four-class classification problem in which the temporal relation between two clinical events is predicted as BEFORE, AFTER, OVERLAP, or NONE. The study centers on long-context transformer architectures for modeling extended event-pair context windows, where temporally relevant evidence may be distributed across multiple sentences or broader surrounding clinical context.

The experimental design emphasizes rigorous subject-level partitioning to prevent patient-level leakage and supports systematic comparison across in-domain evaluation, inter-dataset zero-shot transfer, and inter-dataset adaptation settings. Within this framework, Clinical-Longformer and Clinical-BigBird are treated as the primary long-context models, whereas BERT-base, BioBERT, Bio_ClinicalBERT, and BiomedBERT are included as standard 512-token baseline models for comparative evaluation. The experiments are conducted on prepared temporal relation datasets derived from MIMIC-III and MIMIC-IV, where each sample corresponds to an event-pair instance rather than a complete raw clinical note.

This research is limited to rule-derived event-pair temporal relation classification and does not address full end-to-end clinical information extraction, automatic event detection, temporal expression normalization, manually annotated temporal relation extraction, or complete patient-level timeline generation. Therefore, the scope is specifically to evaluate whether long-context clinical transformers improve temporal relation classification performance and robustness across related but distinct EHR datasets in a silver-label experimental setting.

### 1.2 Motivation and research contributions

Clinical temporal relation extraction remains challenging because the evidence required to determine the temporal ordering of events is frequently not confined to a short textual span. In clinical notes, relevant contextual cues may be distributed across lengthy surrounding contexts, making short-context encoders vulnerable to truncation-related information loss. This problem becomes even more important when models are evaluated across different clinical corpora, where note structure, documentation style, class distribution, and corpus organization may vary. The motivation for this research is therefore twofold: first, to examine whether long-context transformer architectures can better preserve extended event-pair context for temporal relation classification; and second, to determine whether such models remain effective across inter-dataset variation in major EHR corpora.

The major contributions of this research are as follows:

1. A rule-derived clinical temporal relation extraction framework is developed in which the relation between event pairs is modeled as a four-class classification problem with the labels BEFORE, AFTER, OVERLAP, and NONE.
2. Clinical-Longformer and Clinical-BigBird are employed as the principal long-context architectures to capture broader contextual dependencies in extended event-pair contexts. A progressive two-stage training strategy is used, in which the models are first fine-tuned at 1,024 tokens and then continued at 4,096 tokens from the best Stage 1 checkpoint to address GPU memory constraints while preserving longer-range temporal evidence.
3. BERT-base, BioBERT, Bio_ClinicalBERT, and BiomedBERT are incorporated as standard 512-token baseline models to provide comparisons across general-domain, biomedical, and clinical pretraining under the same task formulation, data partitioning strategy, and evaluation protocol.
4. A leakage-free subject-level partitioning strategy is adopted to ensure rigorous evaluation by preventing patient overlap across training, validation, and test sets, with additional note-level overlap checks used to confirm strict partition independence.
5. The framework is evaluated under in-domain, inter-dataset zero-shot, and inter-dataset adaptation settings, enabling systematic assessment of robustness across MIMIC-III and MIMIC-IV.
6. The expanded six-model evaluation provides evidence that the main generalization pattern is consistent across pretrained model families. MIMIC-III-to-MIMIC-IV zero-shot transfer was easier than the reverse direction, while target-domain adaptation improved performance in both directions. Clinical-Longformer and Clinical-BigBird retained the strongest results in the principal robustness-oriented settings, whereas the four standard-context models remained competitive under matched and easier transfer conditions.

#### Paper organization

The remainder of this paper is structured as follows. Section 2 reviews relevant studies on clinical temporal information extraction, temporal relation extraction, and long-context transformer modeling for clinical text. Section 3 describes the proposed experimental framework, including the task definition, data preparation strategy, model architectures, progressive two-stage training procedure, and evaluation protocol. Section 4 presents the experimental results and discussion, with particular emphasis on in-domain performance, inter-dataset transfer, ablation analysis, and robustness interpretation. Section 5 concludes the paper and outlines future research directions.

## 2 Related work

Clinical temporal relation extraction remains an important research problem in clinical natural language processing because meaningful interpretation of patient records depends not only on identifying clinical events, but also on determining their temporal ordering and contextual relationships. Prior work has shown that temporal reasoning in clinical narratives remains challenging because temporal cues are often implicit, distributed across multiple sentences, and sensitive to broader narrative context (3, 8, 16). Recent studies have explored multiple methodological directions for this task. Knez and Žitnik (5) proposed a bimodal architecture that combines textual and knowledge graph information for temporal relation extraction. Chaturvedi et al. (7) introduced a span-based graph transformer approach to capture richer local and global dependencies in clinical temporal relation extraction. Kougia et al. (8) further showed that zero-shot temporal relation extraction in clinical notes remains difficult, especially when consistency and accuracy are jointly evaluated.

Another important direction concerns the extraction of timelines in realistic clinical settings. The 2024 and 2025 ChemoTimelines shared tasks highlighted temporal reasoning as a central component of patient-level chemotherapy timeline construction from clinical notes (13, 14). Similarly, MedTimeline demonstrated an end-to-end hybrid NLP system for extracting timeline information from clinical narratives in a shared-task setting (17). More recent longitudinal temporal modeling work, such as TIMER, has shown that large language models often struggle with temporal reasoning over multi-visit clinical records, although time-aware instruction tuning can improve performance (18). Together, these studies indicate that temporal modeling is increasingly being treated as a broader longitudinal reasoning problem rather than only a local pairwise classification task.

In parallel, the growing use of transformer-based models in clinical NLP has highlighted the limitations of standard short-context encoders for long, extended clinical narratives. Clinical-Longformer and Clinical-BigBird were introduced to process long clinical sequences by extending the input window from 512 to 4,096 tokens through sparse attention mechanisms (10). More broadly, recent work on long clinical documents has emphasized the need for benchmarks and models capable of handling long narrative notes that often exceed the context limits of conventional transformer encoders (19). Related work on LLM-based clinical information extraction and longitudinal clinical reasoning further suggests that robust handling of long and heterogeneous healthcare narratives remains challenging, particularly when temporally distributed evidence must be integrated across records (18, 20).

Recent studies have also emphasized the importance of explainability and trustworthiness in healthcare-oriented language models. The study in (21) discussed the need to make LLM decisions transparent and trustworthy, particularly in healthcare environments where opaque model outputs may reduce accountability, interpretability, and user confidence. Similarly, the healthcare-focused book in (22) addressed transparency, reliability, clinical decision-support systems, explainable clinical NLP, human-centred evaluation, ethical considerations, and integration with clinical workflows. Although the present study evaluates encoder-based temporal-relation classifiers rather than generative LLM-based decision-support systems, these studies remain relevant because clinical NLP models should provide not only accurate predictions but also interpretable evidence and clearly communicated limitations. Future extensions of the present framework may therefore investigate explanation methods that identify the contextual evidence contributing to temporal-relation predictions and assess whether such explanations are clinically meaningful.

Contextualized representations have also been shown to be useful for extracting clinically relevant events and entities from complex biomedical and clinical narratives (23). Prior systematic reviews have further emphasized that temporal relation extraction from clinical free text remains methodologically challenging due to task complexity, candidate-pair generation, dataset imbalance, and limited diversity in evaluation corpora (24). Recent work on large temporal models has also demonstrated that fine-tuning focused on temporal relations can improve temporal relation classification performance across general-domain benchmarks (25). Relative-timeline extraction frameworks have similarly highlighted the importance of structuring clinical events temporally for patient-level timeline analysis (26).

Taken together, prior work has advanced clinical temporal relation extraction, timeline extraction, longitudinal temporal reasoning, and long-context modeling from several complementary directions. However, most existing studies have focused on benchmark-specific settings, timeline reconstruction tasks, or broader temporal reasoning frameworks, with comparatively limited attention to a unified, robust comparison across both MIMIC-III and MIMIC-IV. In particular, there remains limited evidence regarding whether long-context clinical transformers provide measurable advantages over standard-context biomedical encoders for rule-derived event-pair temporal relation classification under in-domain, zero-shot inter-dataset, and adaptation-based transfer settings. Recent research on explainable healthcare LLMs further indicates that robustness-oriented evaluation should eventually be complemented by transparency and clinically meaningful explanations, although the development of an explainability framework is outside the scope of the present study. The present study is therefore positioned as a systematic empirical investigation of long-context vs. standard-context modeling across two major EHR corpora, with particular emphasis on robustness under inter-dataset variation.

## 3 Methodology

### 3.1 Study Overview

**Figure 1:**
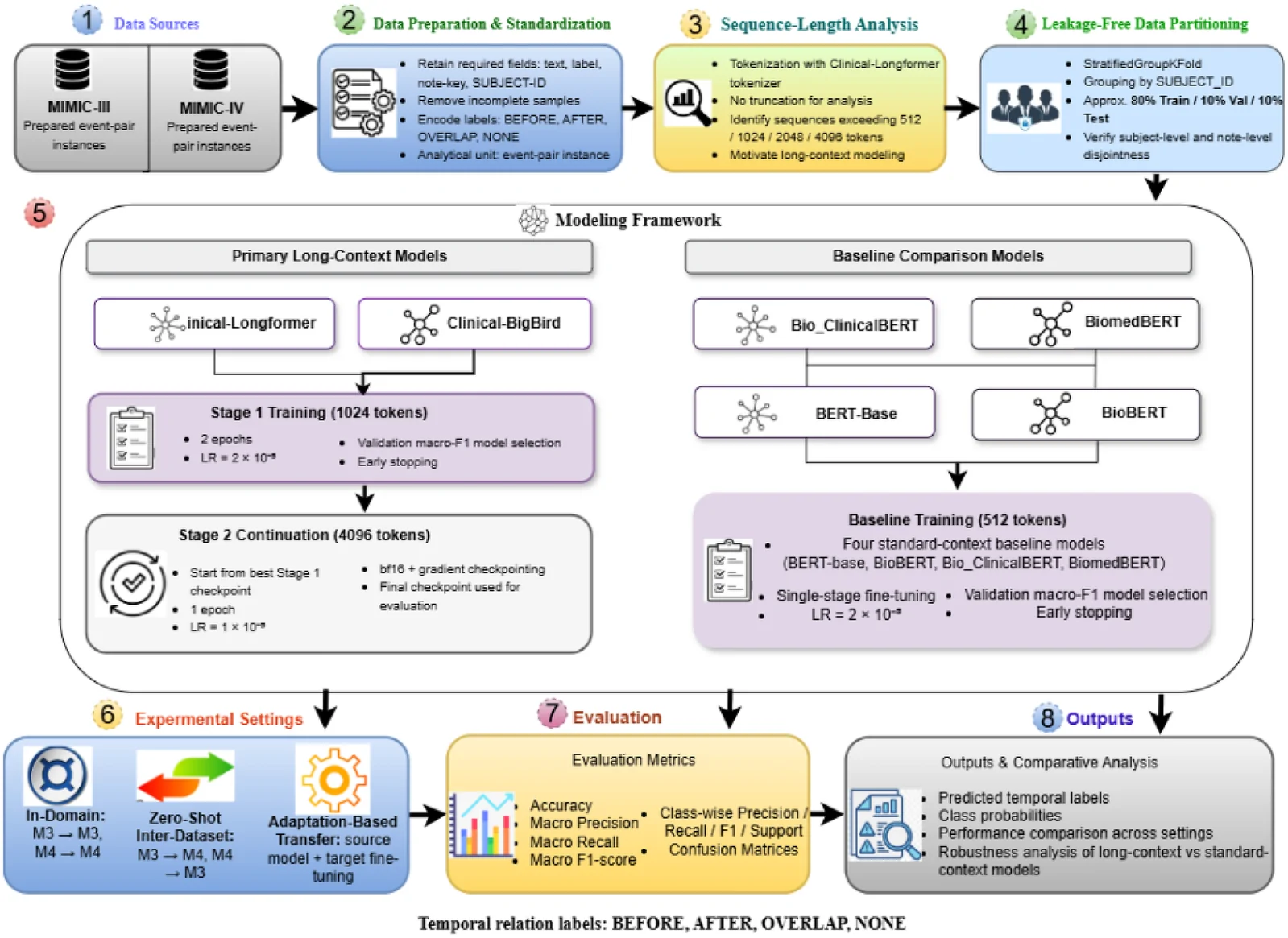
Overall workflow of the proposed long-context clinical temporal relation extraction framework.

This study investigates rule-derived clinical temporal relation extraction from electronic health record (EHR) narratives using transformer-based language models. The task is formulated as a silver-label four-class classification problem with the labels BEFORE, AFTER, OVERLAP, and NONE. The proposed framework uses prepared event-pair datasets derived from MIMIC-III and MIMIC-IV and includes standardized label encoding, sequence-length analysis, leakage-free subject-level partitioning, two-stage long-context model training, and evaluation under in-domain and inter-dataset settings. Figure 1 summarizes the complete methodological workflow.

The central objective of the study is to determine whether long-context clinical transformers provide practical advantages for temporal relation extraction when relevant temporal evidence is distributed across extended event-pair context windows. To contextualize the performance of the primary models, BERT-base, BioBERT, Bio_ClinicalBERT, and BiomedBERT were included as standard-context baseline models under the same experimental protocol. However, the proposed framework itself is centered on Clinical-Longformer and Clinical-BigBird, whereas the baseline models serve as reference systems for comparative evaluation.

### 3.2 Task formulation

Let *x* denote the prepared input text corresponding to an event-pair instance, and let *y* denote the temporal relation label to be predicted. The objective is to assign each instance to one of four rule-derived temporal relation classes. The label space is defined as

y={BEFORE,AFTER,OVERLAP,NONE} (1)

Equation (1) defines the complete label space used throughout the study. BEFORE indicates that the first event mention is resolved to an earlier timestamp than the second; AFTER indicates that the first event mention is resolved to a later timestamp than the second; OVERLAP indicates that both event mentions are resolved to the same calendar date; and NONE denotes event pairs for which a deterministic temporal relation could not be assigned because one or both timestamps could not be resolved. Thus, NONE should be interpreted as a rule-derived unresolved category rather than a manually annotated absence of temporal relation.

Accordingly, the problem is formulated as a silver-label four-class classification task:

$$f_{\theta }(x)\to y,\;y\;\epsilon \;Y$$ (2)

Equation (2) expresses the task formulation adopted in this work. Here, the classifier $f_{\theta }$ maps the prepared event-pair input text to one of the predefined temporal relation labels, thereby enabling consistent learning and evaluation across all models.

### 3.3 Data source and corpus construction

The experiments used deidentified clinical text derived from MIMIC-III and MIMIC-IV, two credentialed-access critical-care EHR resources available through PhysioNet. Access was obtained under the PhysioNet credentialed data-access agreement after completing the required data-privacy training. Because both datasets were deidentified before public release, no additional institutional ethics review was required. No direct patient contact or re-identification was involved. MIMIC-III contains data from more than 40,000 critical-care patients, whereas MIMIC-IV provides a newer organization of clinical data from the same institution. Although the datasets share an institutional origin, they differ in structure, note organization, and documentation characteristics. Their comparison therefore represents inter-version dataset transfer rather than true cross-institution transfer, which may limit generalizability to other healthcare systems.

In the implemented pipeline, the experiments were not performed directly on raw note tables. Instead, prepared temporal relation datasets derived from MIMIC-III and MIMIC-IV were used, in which each row corresponded to a single rule-derived event-pair instance associated with a clinical note. Each instance contained the prepared input text, the silver temporal relation label, a patient identifier (SUBJECT_ID), and a note identifier (note_key). Thus, the analytical unit in this study was the event-pair instance rather than the complete clinical note. For each event pair, the model input text was constructed by extracting a local sentence context window of two sentences before and after the sentences containing the two event mentions, with the two mentions marked using special tokens [E1]/[/E1] and [E2]/[/E2]. This focused context-window approach allows the model to receive local and surrounding temporal evidence without processing the entire discharge note, which may contain thousands of tokens. Although the full note was not used as input, the token-length analysis in Section 3.5 shows that a meaningful proportion of prepared event-pair contexts still exceeded the 512-token limit of standard BERT-style encoders.

The rule-based label-generation pipeline consisted of clinical-note preprocessing, sentence segmentation, temporal-expression detection, timestamp normalization, event-pair construction, and deterministic relation assignment. It was applied to 59,652 MIMIC-III discharge summaries extracted from NOTEEVENTS and 331,793 MIMIC-IV discharge summaries. Before event extraction, MIMIC-III deidentification placeholders of the form [**…**] were removed using a regular-expression pattern. Notes were segmented using the NLTK sent_tokenize function. Temporal expressions were identified using regular-expression patterns covering calendar dates, postoperative day references (POD), hospital day references (HD), day-of-life references (DOL), and relative expressions such as “yesterday” and “tomorrow.” The detected expressions were resolved to absolute datetimes using the dateparser library and available reference timestamps, including ADMITTIME, CHARTDATE, CHARTTIME, and DISCHTIME.

Eligible event pairs were constructed from temporal mentions occurring within a four-sentence sliding window. This threshold was selected because preliminary analysis indicated that most resolvable temporal pairs occurred within this range, balancing the retention of valid pairs against the inclusion of unrelated distant mentions. Labels were assigned by comparing the resolved timestamps: BEFORE when the first event preceded the second, AFTER when it followed the second, OVERLAP when both resolved to the same calendar date, and NONE when either timestamp could not be resolved. To control computational cost, a maximum of 20 temporal mentions and 60 event pairs was retained per note, with random subsampling applied when these limits were exceeded. The fixed random seed used for data partitioning and model training is reported in Section 3.12.

Because automatic timestamp comparison does not guarantee clinical correctness, errors may arise from temporal-expression detection, incomplete event spans, ambiguous temporal anchors, or datetime resolution. The resulting labels were therefore treated as silver labels rather than manually annotated gold-standard labels. A physician separately reviewed 200 event-pair instances to assess the reliability of this supervision, as described in Section 3.15.3.

The prepared MIMIC-III corpus contained 159,542 instances, which were divided into 127,518 training, 15,944 validation, and 16,080 test instances after subject-level grouping. The MIMIC-IV corpus contained 209,482 instances, divided into 166,981 training, 21,006 validation, and 21,495 test instances. Table 1 summarizes these corpus statistics.

**Table 1:** Statistics of the prepared temporal relation datasets used in this study.

Dataset	Raw prepared instances	Train instances	Validation instances	Test instances	Split strategy	Labels
MIMIC-III	159,542	127,518	15,944	16,080	StratifiedGroupKFold with SUBJECT_ID grouping	BEFORE, AFTER, OVERLAP, NONE
MIMIC-IV	209,482	166,981	21,006	21,495	StratifiedGroupKFold with SUBJECT_ID grouping	BEFORE, AFTER, OVERLAP, NONE

The two corpora exhibited different class distributions. In MIMIC-III, BEFORE was the dominant class, representing 62.5% of instances (99,736), followed by NONE at 23.6% (37,590), AFTER at 8.7% (13,948), and OVERLAP at 5.2% (8,268). In MIMIC-IV, NONE was the largest class at 46.4% (97,260), followed by BEFORE at 33.7% (70,606), OVERLAP at 12.4% (25,988), and AFTER at 7.5% (15,628). The underrepresentation of AFTER and OVERLAP motivated the use of class-weighted cross-entropy loss. These differences also introduced a meaningful distributional shift for inter-dataset transfer evaluation.

### 3.4 Data preparation and label encoding

To ensure consistency across datasets and models, the event-pair corpora derived from MIMIC-III and MIMIC-IV were standardized into a common tabular format. The final pipeline required four essential fields: text, label, note_key, and SUBJECT_ID. Samples containing missing values in any of these fields were removed prior to training and evaluation.

Let the dataset be represented as

$$D=\{\left(x_{n},y_{n},s_{n},n_{n}\right)\}\begin{array}{c} N\\ n=1 \end{array}$$ (3)

Equation (3) represents the final dataset structure used in the experiments. Here, $x_{i}$ denotes the prepared text for the *i*-th instance, $y_{i}$ is the temporal relation label, $s_{i}$ is the patient identifier, $m_{i}$ is the note identifier, and *N* is the number of valid instances.

The temporal relation labels were encoded using a unified numerical mapping:

BEFORE=0,AFTER=1,OVERLAP=2,NONE=3 (4)

Equation (4) presents the label-encoding scheme applied across both datasets. This common mapping ensured a consistent learning target for all experimental settings and models.

### 3.5 Sequence-length analysis and motivation for long-context modeling

Although the model inputs consist of focused event-pair contexts rather than complete discharge summaries, some instances exceed the 512-token limit of standard BERT-style encoders. Truncation may remove relevant temporal evidence located outside the immediate event mentions. In the prepared datasets, 9.84% of MIMIC-III instances and 17.78% of MIMIC-IV instances exceeded 512 tokens, motivating the evaluation of long-context models capable of processing up to 4,096 tokens.

Token-length analysis was performed on the prepared event-pair contexts using the Clinical-Longformer tokenizer without truncation. Let $l_{i}$ denote the tokenized length of the *i*-th prepared instance. The mean token length is defined as.

$$\mu_{l}=\;\frac{1}{N}\;\overset{N}{\underset{i=1}{\sum }}\;l_{i}$$ (5)

Equation (5) gives the mean token length over all prepared instances.

The proportion of instances exceeding a specified token limit L is defined as

$$R_{>L}=\;\frac{1}{N}\;\overset{N}{\underset{i=1}{\sum }}\;1(l_{i}>L)$$ (6)

Equation (6) defines the proportion of sequences whose tokenized length exceeds a specified threshold *L*, where 1(⋅) is an indicator function that returns 1 when the tokenized length exceeds L and 0 otherwise. Table 2 summarizes the token-length distributions of the MIMIC-III and MIMIC-IV training corpora.

**Table 2:** Token-length distributions of the MIMIC-III and MIMIC-IV training corpora.

Statistic	MIMIC-III	MIMIC-IV
Mean tokens	255.6	339.5
Median tokens	171.0	211.0
75th percentile	235.0	345.0
90th percentile	500.0	822.0
95th percentile	790.0	1,058.0
99th percentile	1,371.0	1,456.0
Maximum tokens	6,412	7,710
% exceeding 512 tokens	9.84%	17.78%
% exceeding 1,024 tokens	2.45%	5.62%
% exceeding 2,048 tokens	0.41%	0.17%
% exceeding 4,096 tokens	0.04%	0.01%

MIMIC-IV contained longer prepared contexts on average, with a mean length of 339.5 tokens compared with 255.6 tokens for MIMIC-III. Its median, 90th-percentile, and 95th-percentile lengths were also higher, indicating a heavier sequence-length distribution. Although relatively few instances exceeded 2,048 or 4,096 tokens, the absolute number remained meaningful because of the large corpus sizes. For example, approximately 3,124 MIMIC-III training instances exceeded 1,024 tokens.

These findings support the use of Clinical-Longformer and Clinical-BigBird as the primary models. Their 4,096-token capacity reduces the truncation of extended temporal evidence while retaining compatibility with the shorter event-pair contexts that constitute most instances.

### 3.6 Leakage-free data partitioning

To ensure rigorous evaluation, the datasets were partitioned using StratifiedGroupKFold with SUBJECT_ID as the grouping variable. This design was necessary because multiple event-pair instances may originate from the same patient, and distributing them across training, validation, and test subsets could introduce patient-level leakage.

The partitioning was conducted in two stages. First, a five-fold StratifiedGroupKFold procedure was used to allocate approximately 80% of the subject groups to the training set and the remaining 20% to a temporary subset. Second, a two-fold StratifiedGroupKFold procedure divided the temporary subset equally into validation and test sets. Both partitioning stages were performed with shuffling enabled and a fixed random state of 42.

Let $S_{\mathrm{train}}$, $S_{\mathrm{val}}$, and $S_{\mathrm{test}}$ denote the subject sets corresponding to the three partitions. The subject-disjoint splitting constraint is expressed as

$$s_{train}\cap \;s_{val}=\emptyset ,\;\;s_{train}\;\cap \;s_{test}=\emptyset ,\;\;s_{val\;}\cap \;s_{test}=\emptyset$$ (7)

Equation (7) states the subject-level disjointness requirement imposed during partitioning.

The approximate split proportions are defined as

$$|D_{train}|\approx 0.8N,\;|D_{val}|\approx 0.1N\;|D_{test}|\approx 0.1N\;\;$$ (8)

Equation (8) indicates the approximate 80:10:10 partition ratio.

Additional note-level overlap checks were performed after partitioning to confirm strict separation between splits. For both MIMIC-III and MIMIC-IV, zero overlap was observed between the training and validation note groups. The resulting grouped splits also closely preserved class distributions across partitions, indicating that the stratified grouping procedure maintained label balance while preventing note-level leakage. The resulting splits were saved and reused consistently across all experimental stages to ensure reproducibility.

### 3.7 Input representation

Each event-pair instance was represented using the prepared text field contained in the dataset, which consists of a local sentence context window of two sentences surrounding each event mention, with the two event mentions marked using [E1]/[/E1] and [E2]/[/E2] special tokens. The pipeline directly tokenized the context-window text using the pretrained tokenizer for the selected backbone model.

Let the tokenized sequence be denoted by

$$t=[t_{1},t_{2}\ldots ,t_{T}]$$ (9)

Equation (9) denotes the token-sequence representation used for model input, where *T* is the original tokenized length before truncation or padding.

For a model-specific maximum sequence length $L_{\max}$, the effective encoded length is

$$T^{\prime }=\min(T,\;\;L_{max})$$ (10)

Equation (10) defines the effective sequence length after truncation. For Clinical-Longformer and Clinical-BigBird, the first training stage was used $L_{\max}=1,024$, followed by continuation training with $L_{\max}=4,096$. For BERT-base, BioBERT, Bio_ClinicalBERT and BiomedBERT, $L_{\max}=512$. During tokenization, truncation was applied according to the model-specific limit, attention masks were generated automatically, and dynamic padding was performed at the batch level, with padding to multiples of 8 for computational efficiency.

### 3.8 Primary long-context models

#### 3.8.1 Clinical-Longformer

Clinical-Longformer was selected as one of the two principal models because it is designed for efficient processing of long clinical sequences. Unlike standard transformers, which rely on full self-attention across the entire sequence, Longformer uses sparse attention mechanisms that enable efficient long-sequence encoding while preserving long-range dependencies. In this study, the model was trained progressively, first at 1,024 tokens and then at 4,096 tokens, to retain longer-range temporal cues that may be lost under shorter input limits.

#### 3.8.2 Clinical-BigBird

Clinical-BigBird was adopted as the second primary model because it also supports efficient long-sequence modeling through sparse attention. BigBird combines global attention, local-window attention, and random attention, allowing it to capture both local and long-range contextual dependencies while remaining computationally tractable on lengthy inputs. In the final reported experiments, Clinical-BigBird was configured with a maximum input length of 4,096 tokens. For instances whose tokenized length fell below BigBird's minimum block-sparse threshold, determined by its block size and random block configuration, the library automatically fell back to full self-attention. This behavior did not affect correctness, but it means that block-sparse attention was not uniformly applied across all instances.

### 3.9 Baseline comparison models

To contextualize the effectiveness of the proposed long-context framework, BERT-base, BioBERT, Bio_ClinicalBERT, and BiomedBERT were included as standard-context baseline models. These models were selected because they represent general-domain, biomedical-domain, and clinical-domain pretrained transformer encoders and provide strong short-context references for evaluating whether broader contextual coverage yields measurable benefits in clinical temporal relation extraction. Together, the four baseline models provide representative standard-context comparisons under the same task formulation, partitioning strategy, optimization procedure, and evaluation protocol as the primary long-context models, but with a maximum sequence length of 512 tokens. Their role in this study is comparative rather than central to the proposed framework.

### 3.10 Classification layer

Each model was implemented as a pretrained transformer encoder followed by a task-specific linear classification head. Let *h* denote the final contextual representation used for prediction. The class logits are computed as.

z=Wh+b (11)

Equation (11) computes the class logits, where *W* and b are trainable parameters of the classification head.

The logits are transformed into class probabilities by the softmax function:.

$$p_{c}=\frac{\exp(z_{c})}{\sum_{k=1}^{4}\;\exp(z_{k})},\;c\in \{1,2,3,4\}$$ (12)

Equation (12) defines the softmax-based probability assigned to each class, where $p_{c}$ denotes the predicted probability of class *c* and K=4.

The final predicted label is obtained as.

$$\hat{y}=\arg\underset{c}{\max}\;p_{c}.$$ (13)

Equation (13) defines the final prediction rule. This uniform output-layer design ensures that performance differences primarily reflect the contextual encoding capacity of the underlying backbone models.

### 3.11 Loss function

Because the temporal relation labels are imbalanced, training was performed using a class-weighted cross-entropy loss. In MIMIC-III, the imbalance is severe, with BEFORE comprising 62.5% of instances and OVERLAP comprising only 5.2%, yieldi a maximum-to-minimum class ratio of 12.1. In MIMIC-IV, the imbalance is moderate at 6.2, with NONE comprising 46.4% and AFTER comprising 7.5% of instances. These imbalances directly motivated the use of normalized inverse-frequency class weighting during training.

Let $n_{c}$ denote the number of training samples in class *c*, *N* the total number of training samples, and K=4 the number of classes. The raw class weight is defined as

$$\tilde{w_{c}}=\;\frac{N}{k\;.\;\;n_{c}}\;$$ (14)

Equation (14) defines the raw class weight assigned to class *c*, assigning relatively larger weights to underrepresented classes.

To stabilize the scale of the weights, the normalized class weight is computed as

$$w_{c}=\;\frac{\tilde{w_{c}}}{\frac{1}{k}\;\sum_{k=1}^{k}\;\tilde{w_{k}}}$$ (15)

Equation (15) presents the normalized class-weight formulation. This normalization preserves the relative weighting pattern while keeping the overall scale controlled.

For a sample with an assigned label *y*, the weighted cross-entropy loss is

$$L_{CE}=\;-w_{y}\;\log\;(\;p_{y})$$ (16)

Equation (16) gives the weighted cross-entropy loss for a single sample, increasing the influence of minority-class errors during optimization.

For a mini-batch of size *B*, the final objective becomes

$$L=\;\frac{1}{B}\;\overset{B}{\underset{b=1}{\sum }}\;L_{CE}^{\left(b\right)}$$ (17)

Equation (17) defines the mini-batch training objective as the average of weighted cross-entropy values across the batch.

### 3.12 Training configuration

All models were fine-tuned using the Hugging Face Transformers framework. Clinical-Longformer and Clinical-BigBird followed the two-stage procedure described in Section 3.12.1. BERT-base, BioBERT, Bio_ClinicalBERT, and BiomedBERT were trained in a single stage with a maximum sequence length of 512 tokens, a training batch size of 8, an evaluation batch size of 16, and two gradient-accumulation steps.

The models were initialized from the following publicly available pretrained checkpoints: yikuan8/Clinical-Longformer, yikuan8/Clinical-BigBird, google-bert/bert-base-uncased, dmis-lab/biobert-base-cased-v1.2, emilyalsentzer/Bio_ClinicalBERT, and microsoft/BiomedNLP-BiomedBERT-base-uncased-abstract-fulltext.

For reproducibility, a fixed random seed of 42 was applied to Python, NumPy, PyTorch CPU and CUDA generators, and the Hugging Face training configuration. PyTorch cuDNN deterministic mode was enabled, while cuDNN benchmarking was disabled. The same saved subject-disjoint partitions were reused across all experiments. Each model configuration was trained once using the fixed seed; repeated training with multiple independent seeds was not performed.

The learning rate for standard in-domain training and Stage 1 of the long-context models was defined as

$$\eta_{in}=2\times 10^{-5}$$ (18)

Equation (18) specifies the learning rate used for standard in-domain model training.

A reduced learning rate was used for the 4,096-token continuation stage and adaptation-based transfer:

$$\eta_{adapt}=1\times 10^{-5}$$ (19)

Equation (19) specifies the reduced learning rate used during both the 4,096-token continuation phase and inter-dataset adaptation.

Label smoothing was not applied during training, as the class-weighted loss was used to address class imbalance without additional label-level regularization. The warm-up ratio was fixed at

$$\gamma_{warmup}=0.06\;$$ (20)

Equation (20) presents the warm-up ratio setting adopted across all training stages.

For validation-based training, model evaluation and checkpoint saving were performed every 1,000 steps. The checkpoint with the highest validation macro-F1 score was reloaded at the end of training, with early stopping applied using a patience of three evaluation rounds. The selected model and tokenizer were saved for subsequent evaluation. For the four standard-context baselines, the validation-selected checkpoint was used for final test evaluation.

#### 3.12.1 Two-stage training for long-context models

Clinical-Longformer and Clinical-BigBird were trained in two stages because direct fine-tuning with 4,096-token inputs required substantially greater GPU memory.

In Stage 1, both models were fine-tuned from their pretrained checkpoints with a maximum sequence length of 1,024 tokens for two epochs. The training batch size was 2, the evaluation batch size was 4, and four gradient-accumulation steps produced an effective batch size of 8. Model selection was based on validation macro-F1. Evaluation and checkpoint saving were performed every 1,000 steps, and early stopping was applied with a patience of three evaluation rounds. The checkpoint with the highest validation macro-F1 was retained for Stage 2.

In Stage 2, the selected Stage 1 checkpoint was further fine-tuned with a maximum sequence length of 4,096 tokens for one epoch using the reduced learning rate $\eta_{\mathrm{adapt}}=1\times 10^{-5}$. A training batch size of 1 and 32 gradient-accumulation steps produced an effective batch size of 32. Bfloat16 precision and gradient checkpointing were enabled to reduce memory usage. Because intermediate validation and early stopping were not performed during this stage, the final-epoch checkpoint was retained.

The Stage 2 checkpoint was used for in-domain testing and direct zero-shot evaluation on the other corpus. For adaptation-based transfer, the corresponding source-domain Stage 2 checkpoint was fine-tuned for one additional epoch on the target-domain training set, and the resulting checkpoint was used for target-domain testing.

This progressive strategy initializes the 4,096-token stage from a trained 1,024-token checkpoint, reducing the need to train from scratch at the longer sequence length. However, because Stage 2 introduces both additional optimization and a larger context window, the present design cannot isolate the independent contribution of the 4,096-token context. A direct single-stage 4,096-token experiment initialized from the original pretrained checkpoint would be required to separate these effects.

### 3.13 Computational environment and efficiency measurement

All experiments were conducted in Google Colab using an NVIDIA A100-SXM4-40GB GPU. The GPU's native bfloat16 support was used during the 4,096-token training stage to reduce memory consumption.

Computational efficiency was evaluated using the final saved checkpoints on the same GPU. The benchmark included 1,000 instances from the MIMIC-IV test set with a batch size of one. After 50 warm-up iterations, three timed repetitions were performed over the complete benchmark set. CPU tokenization time was excluded, whereas CPU-to-GPU transfer and model forward-pass time were included. The recorded measures were parameter count, mean inference latency, throughput, and peak allocated inference GPU memory

Exact wall-clock training times were not automatically retained. Based on author observations, Clinical-Longformer and Clinical-BigBird each required approximately 8–9 h for the complete two-stage procedure, whereas Bio_ClinicalBERT and BiomedBERT each required approximately 5–6 h for single-stage training. These values are reported as approximate ranges rather than precise runtime measurements. Peak training-memory values were also unavailable; therefore, the reported GPU-memory measurements refer only to inference.

### 3.14 Experimental settings

To comprehensively evaluate model robustness, three complementary experimental settings were considered.

#### 3.14.1 In-domain evaluation

In the in-domain setting, training, validation, and testing were performed within the same dataset version:

$$D_{train}^{M3}\to \;D_{test}^{M3}\;\;\;\;\;D_{train}^{M4}\to \;D_{test}^{M4}$$ (21)

Equation (21) defines the in-domain evaluation setting. This setting measures dataset-specific performance under leakage-free partitioning.

#### 3.14.2 Zero-shot inter-dataset evaluation

In the zero-shot setting, a model trained on one MIMIC version was directly evaluated on the other without additional fine-tuning:

$$D_{train}^{M3}\to \;D_{test}^{M4}\;\;\;D_{train}^{M4}\to \;D_{test}^{M3}$$ (22)

Equation (22) defines the zero-shot inter-dataset evaluation protocol, which assesses the robustness of learned temporal representations to inter-dataset variation.

#### 3.14.3 Adaptation-based inter-dataset evaluation

In the adaptation setting, a model trained on one dataset was further fine-tuned on the target dataset using the reduced learning rate $\eta_{\mathrm{adapt}}$:

$$\theta_{M3\to M4}^{*}=Adapt\;(\theta_{M3}^{*},D_{train}^{M4})$$ (23)

Equation (23) represents adaptation-based transfer from MIMIC-III to MIMIC-IV.

$$\theta_{M4\to M3}^{*}=Adapt\;(\theta_{M4}^{*},D_{train}^{M3})$$ (24)

Equation (24) represents adaptation-based transfer from MIMIC-IV to MIMIC-III. Together, these formulations describe the bidirectional adaptation setting used for inter-dataset evaluation.

### 3.15 Evaluation metrics

Performance was assessed using accuracy, macro precision, macro recall, macro F1-score, and class-wise precision, recall, F1-score, and support. Let $TP_{c}$, $FP_{c}$, and $FN_{c}$ denote the true positives, false positives, and false negatives for class *c*.

The class-wise precision was computed as

$$Precision_{c}=\;\frac{TP_{c}}{TP_{c}+\;FP_{c}}$$ (25)

Equation (25) defines class-wise precision for the temporal relation class *c*.

The class-wise recall was computed as

$$Recall_{c}=\;\frac{TP_{c}}{TP_{c}+\;FN_{c}}$$ (26)

Equation (26) defines class-wise recall for the temporal relation class *c*.

The class-wise F1-score was computed as

$$F1_{c}=\;\frac{2\;.\;\;Precision_{c}\;.\;\;Recall_{c}}{Precision_{c}\;+\;Recall_{c}}$$ (27)

Equation (27) defines the class-wise F1-score as the harmonic mean of precision and recall.

Macro-averaged precision was obtained as

$$Macro\;Precision=\;\frac{1}{K}\;\overset{K}{\underset{c=1}{\sum }}\;Precision_{c}$$ (28)

Equation (28) gives the macro-averaged precision across all classes.

Macro-averaged recall was obtained as

$$Macro\;Recall=\;\frac{1}{K}\;\overset{K}{\underset{c=1}{\sum }}\;Recall_{c}$$ (29)

Equation (29) gives the macro-averaged recall across all classes.

Macro-averaged F1-score was obtained as

$$Macro\;F1=\;\frac{1}{K}\;\overset{K}{\underset{c=1}{\sum }}\;F1_{c}$$ (30)

Equation (30) gives the macro-averaged F1-score across the label space.

Overall accuracy was computed as

$$Accuracy=\;\frac{\sum_{c=1}^{k}\;TP_{c}}{N_{test}}$$ (31)

Equation (31) defines overall classification accuracy on the test set. Because the task is imbalanced, macro F1-score was used as the primary evaluation metric. Confusion matrices were additionally used to examine class-specific misclassification patterns.

#### 3.15.1 Statistical significance analysis

Statistical significance testing used saved test-set predictions from Clinical-Longformer, Clinical-BigBird, Bio_ClinicalBERT, and BiomedBERT evaluated on the same subject-disjoint instances. Pairwise correctness differences were assessed using McNemar's test. The exact binomial form was used for small discordant counts, whereas the continuity-corrected chi-square approximation was used for larger counts.

Five comparisons were conducted in each of the six experimental settings: Clinical-Longformer vs. Clinical-BigBird and each long-context model vs. Bio_ClinicalBERT and BiomedBERT. Holm–Bonferroni correction was applied across the resulting 30 comparisons to control the family-wise error rate at α=0.05. A difference was considered significant when the Holm-adjusted *p*-value was below 0.05. BERT-base and BioBERT were included only in the expanded predictive evaluation and were not part of this primary McNemar family. The four Stage 1 vs. Stage 1 + 2 ablation comparisons were corrected separately.

Subject-level paired bootstrap resampling with 1,000 replicates was additionally used to estimate 95% confidence intervals and bootstrap *p*-values for the macro-F1 difference between the strongest long-context and strongest standard-context model in each setting. Subject identifiers were resampled with replacement to preserve dependence among event-pair instances from the same patient. A difference was considered statistically supported when its confidence interval excluded zero. These six bootstrap comparisons were conducted separately from the 30 Holm-corrected McNemar comparisons. Since neither BERT-base nor BioBERT was the strongest standard-context model in any setting, their addition did not alter the selected bootstrap comparisons.

#### 3.15.2 Qualitative error-analysis protocol

A structured qualitative analysis was conducted on 120 misclassified instances to examine error sources beyond aggregate metrics and confusion matrices. The sample included ten errors from each combination of the two long-context models and six experimental settings, providing balanced coverage across models, evaluation conditions, and observed true-label-to-predicted-label confusion types.

Each instance was reviewed using the event-pair context, silver label, model prediction, and marked event spans. The analysis considered possible silver-label noise, abbreviation or formatting problems, sentence-segmentation errors, ambiguous temporal anchors, competing temporal expressions, same-day or time-granularity ambiguity, and other contextual difficulties.

Each instance was assigned one mutually exclusive primary error category for quantitative aggregation. Additional issues were recorded as secondary observations but were not double-counted. Therefore, the primary-category counts summed to all 120 reviewed errors.

Because the sample was drawn specifically from model misclassifications, the resulting proportions describe only the reviewed error set and not the overall label-noise rate of the complete datasets. This analysis was conducted separately from the physician audit described in Section 3.15.3.

#### 3.15.3 Physician review of silver-label reliability

To assess the reliability of the rule-derived silver labels, a physician manually reviewed 200 event-pair instances from the prepared MIMIC-derived datasets. For each instance, the physician examined the event-pair context and determined whether the original rule-derived temporal-relation label was correct or incorrect. This physician review was conducted separately from the qualitative review of model misclassifications.

Physician agreement was calculated as the number of silver labels judged correct divided by the total number of reviewed instances. The observed label-disagreement rate was calculated as the number of silver labels judged incorrect divided by the total number of reviewed instances. Wilson 95% confidence intervals were calculated for both proportions. Because the clinical review covered a limited sample, the resulting disagreement rate was interpreted as the observed noise rate within the reviewed sample rather than as an exact population-wide estimate for the complete datasets.

### 3.16 Proposed algorithm

The overall workflow of the proposed long-context temporal relation extraction framework is summarized in Algorithm 1 and illustrated in Figure 1.

Algorithm 1. **Proposed long-context temporal relation extraction framework**

Input: Prepared MIMIC-III and MIMIC-IV event-pair datasets; primary model set M_p_ = {Clinical-Longformer, Clinical-BigBird}

Output: Trained long-context models, predicted temporal labels, class probabilities, evaluation metrics, and confusion matrices

1. Load the prepared MIMIC-III and MIMIC-IV event-pair datasets.
2. Standardize the labels into BEFORE, AFTER, OVERLAP, and NONE.
3. Remove incomplete samples and retain the required columns: text, label, note_key, and SUBJECT_ID.
4. Compute token-length statistics on the prepared text without truncation, reporting proportions exceeding thresholds of 512, 1,024, 2,048, and 4,096 tokens, as summarized in Table 2.
5. Partition each dataset into training, validation, and test subsets using StratifiedGroupKFold with SUBJECT_ID-based grouping, and save the partitions for consistent reuse across all experimental stages.
6. Verify the absence of patient-level and note-level overlap across partitions.
7. Compute class weights from the training-label distribution using the normalized inverse-frequency formulation.
8. Stage 1–1,024-token training: For each model $m\in M_{p}$, fine-tune from the pretrained checkpoint at maximum sequence length of 1,024 for 2 epochs using $\eta_{\mathrm{in}}=2\times 10^{-5}$, with validation-based model selection and early stopping; save the best checkpoint.
9. Stage 2–4,096-token continuation: For each model $m\in M_{p}$, continue fine-tuning from the best Stage 1 checkpoint at maximum sequence length 4,096 for 1 epoch using $\eta_{\mathrm{adapt}}=1\times 10^{-5}$, bf16 precision, gradient checkpointing, and an effective batch size of 32; save the final epoch checkpoint for all subsequent evaluation.
10. Perform in-domain experiments on MIMIC-III and MIMIC-IV using the Stage 2 checkpoints.
11. Perform zero-shot inter-dataset evaluation for MIMIC-III to MIMIC-IV and MIMIC-IV to MIMIC-III using the Stage 2 checkpoints without additional fine-tuning.
12. Perform adaptation-based inter-dataset transfer by further fine-tuning the source-trained Stage 2 model on the target-domain training split using $\eta_{\mathrm{adapt}}=1\times 10^{-5}$ for 1 epoch.
13. Evaluate all models using accuracy, macro F1-score, macro precision, macro recall, class-wise metrics, and confusion matrices.
14. Compare the effectiveness of the proposed long-context framework against BERT-base, BioBERT, Bio_ClinicalBERT and BiomedBERT under the same evaluation protocol.

### 3.17 Reproducibility and code availability

The code for preprocessing, temporal-expression detection, timestamp resolution, silver-label generation, subject-disjoint partitioning, model training, transfer evaluation, statistical testing, ablation analysis, and computational-efficiency evaluation is publicly available at https://github.com/swethpaddu/clinical-temporal-relation-extraction. The repository contains two documented notebooks and a README describing the execution order, data-access requirements, and procedures for reconstructing the derived datasets. To comply with the PhysioNet data-use agreement, the repository excludes raw MIMIC notes, patient and note identifiers, prepared event-pair text, derived datasets, physician-review worksheets, restricted prediction files, and notebook outputs containing clinical information. Credentialed MIMIC users can reproduce the datasets and experiments by following the provided preprocessing instructions.

## 4 Results

### 4.1 Overall performance across All settings

**Figure 2:**
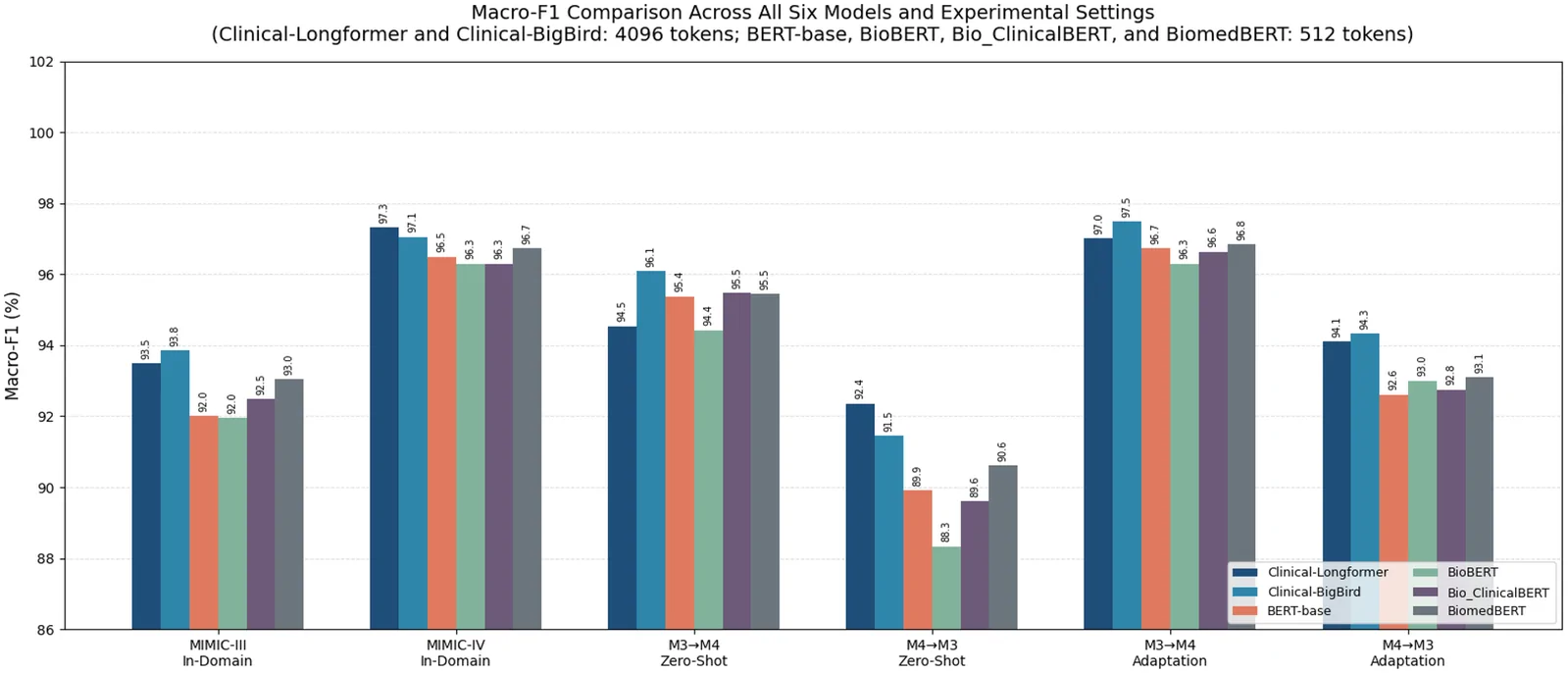
Macro-F1 comparison across all models and experimental settings.

This section presents the quantitative performance of the proposed framework under three evaluation settings: in-domain evaluation, zero-shot inter-dataset transfer, and adaptation-based inter-dataset transfer. The long-context models, Clinical-Longformer and Clinical-BigBird, were evaluated using the final 4,096-token checkpoints obtained through the two-stage training procedure, whereas the baseline models, BERT-base, BioBERT, Bio_ClinicalBERT, and BiomedBERT, were evaluated at a maximum sequence length of 512 tokens. Table 3 summarizes the performance of all six models across the six experimental conditions. Figure 2 provides a visual comparison of macro-F1 across all models and settings.

**Table 3:** Overall performance of all models across all experimental settings.

Setting	Model	Accuracy (%)	Macro-precision (%)	Macro-recall (%)	Macro-F1 (%)
MIMIC-III in-domain	Clinical-Longformer	96.45	95.05	92.10	93.50
MIMIC-III in-domain	Clinical-BigBird	96.69	95.07	92.72	93.85
MIMIC-III in-domain	Bio_ClinicalBERT	95.68	93.30	91.87	92.48
MIMIC-III in-domain	BiomedBERT	96.07	94.45	91.84	93.05
MIMIC-III in-domain	BERT-base	95.50	92.98	91.20	92.01
MIMIC-III in-domain	BioBERT	95.50	92.85	91.20	91.95
MIMIC-IV in-domain	Clinical-Longformer	98.55	97.30	97.34	97.32
MIMIC-IV in-domain	Clinical-BigBird	98.41	97.10	97.04	97.06
MIMIC-IV in-domain	Bio_ClinicalBERT	97.77	96.08	96.52	96.30
MIMIC-IV in-domain	BiomedBERT	98.09	96.63	96.84	96.73
MIMIC-IV in-domain	BERT-base	97.82	96.74	96.26	96.50
MIMIC-IV in-domain	BioBERT	97.73	96.39	96.20	96.30
M3 → M4 zero-shot	Clinical-Longformer	97.24	95.39	93.81	94.54
M3 → M4 zero-shot	Clinical-BigBird	97.95	96.29	95.92	96.10
M3 → M4 zero-shot	Bio_ClinicalBERT	97.14	95.11	95.88	95.48
M3 → M4 zero-shot	BiomedBERT	97.20	95.62	95.28	95.45
M3 → M4 zero-shot	BERT-base	97.20	94.91	95.90	95.38
M3 → M4 zero-shot	BioBERT	96.53	94.02	94.89	94.43
M4 → M3 zero-shot	Clinical-Longformer	95.85	93.86	90.98	92.36
M4 → M3 zero-shot	Clinical-BigBird	95.16	92.94	90.08	91.45
M4 → M3 zero-shot	Bio_ClinicalBERT	93.32	91.61	87.85	89.61
M4 → M3 zero-shot	BiomedBERT	94.37	92.44	88.95	90.61
M4 → M3 zero-shot	BERT-base	93.71	92.52	87.60	89.91
M4 → M3 zero-shot	BioBERT	92.99	89.94	86.95	88.33
M3 → M4 adaptation	Clinical-Longformer	98.42	97.18	96.85	97.01
M3 → M4 adaptation	Clinical-BigBird	98.65	97.50	97.48	97.49
M3 → M4 adaptation	Bio_ClinicalBERT	97.89	97.02	96.29	96.64
M3 → M4 adaptation	BiomedBERT	98.07	97.04	96.66	96.85
M3 → M4 adaptation	BERT-base	98.05	96.79	96.68	96.73
M3 → M4 adaptation	BioBERT	97.74	96.27	96.34	96.30
M4 → M3 adaptation	Clinical-Longformer	96.83	95.54	92.80	94.11
M4 → M3 adaptation	Clinical-BigBird	96.96	95.73	93.06	94.34
M4 → M3 adaptation	Bio_ClinicalBERT	95.83	93.52	92.23	92.75
M4 → M3 adaptation	BiomedBERT	96.09	94.37	91.97	93.10
M4 → M3 adaptation	BERT-base	95.91	93.91	91.50	92.61
M4 → M3 adaptation	BioBERT	96.06	94.47	91.74	93.00

The results indicate that model behavior varies across evaluation settings, rather than a single architecture dominating across all conditions. In the matched in-domain evaluation, all models achieved strong performance on both corpora. Under zero-shot transfer, performance was strongly affected by transfer direction, with M3 → M4 consistently easier than M4 → M3. In adaptation-based transfer, additional fine-tuning on the target-domain training split improved performance across all models, with the strongest transfer observed in the two adaptation settings. Across all six experimental settings, either Clinical-Longformer or Clinical-BigBird achieved the highest macro-F1. The additional BERT-base and BioBERT experiments reproduced the same generalization pattern, showing stronger M3 → M4 zero-shot performance than M4 → M3 performance and improvements in both directions after target-domain adaptation.

Because the temporal relation labels were generated using a deterministic rule-based timestamp-resolution pipeline, these results should be interpreted as performance on large-scale silver-label temporal relation classification rather than as direct equivalence to expert-annotated clinical temporal reasoning. Therefore, the high in-domain scores indicate that the models learned the rule-derived temporal label structure effectively under matched corpus conditions.

The macro-F1 values are further visualized in Figure 2. The grouped comparison shows that a long-context model achieved the highest macro-F1 in every experimental setting, whereas the four standard-context baselines remain strong in some matched and less challenging conditions.

### 4.2 In-domain evaluation results

In the in-domain setting, all six models performed strongly on both MIMIC-III and MIMIC-IV, indicating that rule-derived temporal relation classification is highly learnable when training and testing are conducted within the same corpus. On MIMIC-III, Clinical-BigBird achieved the highest macro-F1 of 93.85%, followed by Clinical-Longformer at 93.50%, BiomedBERT at 93.05%, Bio_ClinicalBERT at 92.48%, BERT-base at 92.01%, and BioBERT at 91.95%. The relatively small margins among the models suggest that all six architectures learned effective temporal representations under matched MIMIC-III conditions.

On MIMIC-IV, Clinical-Longformer achieved the strongest overall performance, with an accuracy of 98.55% and a macro-F1 of 97.32%, followed by Clinical-BigBird at 97.06%, BiomedBERT at 96.73%, BERT-base at 96.50%, and Bio_ClinicalBERT and BioBERT at 96.30% each. Although the long-context models achieved the two highest macro-F1 scores, the four standard-context baselines also remained competitive under matched MIMIC-IV conditions. This indicates that broader contextual coverage provided a measurable advantage, while 512-token models still learned the rule-derived temporal relations effectively within the same corpus.

### 4.3 Zero-shot inter-dataset transfer results

In the zero-shot setting, a clear directional asymmetry was observed across all models. Although MIMIC-III and MIMIC-IV originate from the same institution, the two corpora differ substantially in documentation period, note organization, and class distribution, with MIMIC-IV containing a higher proportion of longer, more carefully prepared event-pair contexts, including 5.62% exceeding 1,024 tokens, compared with 2.45% for MIMIC-III. The class balance also differs markedly, with NONE comprising 46.4% of MIMIC-IV instances compared with 23.6% in MIMIC-III. The observed directional asymmetry in zero-shot transfer performance provides empirical evidence that the two corpora present different distributional characteristics despite sharing an institutional origin. Transfer from MIMIC-III to MIMIC-IV was consistently easier than the reverse, indicating that the two corpora differ in ways that meaningfully affect cross-dataset generalization.

For M3 → M4 zero-shot transfer, Clinical-BigBird achieved the highest macro-F1 of 96.10%, followed by Bio_ClinicalBERT at 95.48%, BiomedBERT at 95.45%, BERT-base at 95.38%, Clinical-Longformer at 94.54%, and BioBERT at 94.43%. The competitive performance of the standard-context baselines in this direction suggests that representations learned from MIMIC-III transfer reasonably well to MIMIC-IV even without target-domain refinement. Nevertheless, Clinical-BigBird remained the best-performing model in this setting.

For the more difficult M4 → M3 direction, Clinical-Longformer achieved the highest macro-F1 of 92.36%, followed by Clinical-BigBird at 91.45%, BiomedBERT at 90.61%, BERT-base at 89.91%, Bio_ClinicalBERT at 89.61%, and BioBERT at 88.33%. In this setting, the strongest long-context model exceeded the strongest standard-context baseline by 1.75 percentage points in macro-F1. These findings suggest that broader contextual modeling can improve robustness under more challenging inter-dataset variation, particularly when temporal evidence must generalize across different documentation styles and corpus structures. The BERT-base and BioBERT results further confirm that the directional transfer asymmetry is consistent across additional pretrained model families.

### 4.4 Adaptation-based transfer results

In the adaptation setting, further fine-tuning the source-trained models on the target-domain training split produced the strongest transfer results in both directions. For M3 → M4 adaptation, Clinical-BigBird achieved the highest macro-F1 of 97.49%, followed by Clinical-Longformer at 97.01%, BiomedBERT at 96.85%, BERT-base at 96.73%, Bio_ClinicalBERT at 96.64%, and BioBERT at 96.30%. In this direction, both long-context models outperformed all four standard-context baselines.

For M4 → M3 adaptation, Clinical-BigBird again achieved the strongest result with a macro-F1 of 94.34%, followed closely by Clinical-Longformer at 94.11%, BiomedBERT at 93.10%, BioBERT at 93.00%, Bio_ClinicalBERT at 92.75%, and BERT-base at 92.61%. Compared with the corresponding zero-shot results, adaptation improved performance across all models, with particularly noticeable gains in the more difficult M4 → M3 direction. Overall, these results indicate that target-domain refinement substantially mitigates cross-corpus degradation and that long-context models remain highly competitive under adaptation-based transfer. The additional BERT-base and BioBERT results confirm that the benefits of target-domain adaptation are consistent across different pretrained model families.

### 4.5 Long-context vs. standard-context comparison

**Figure 3:**
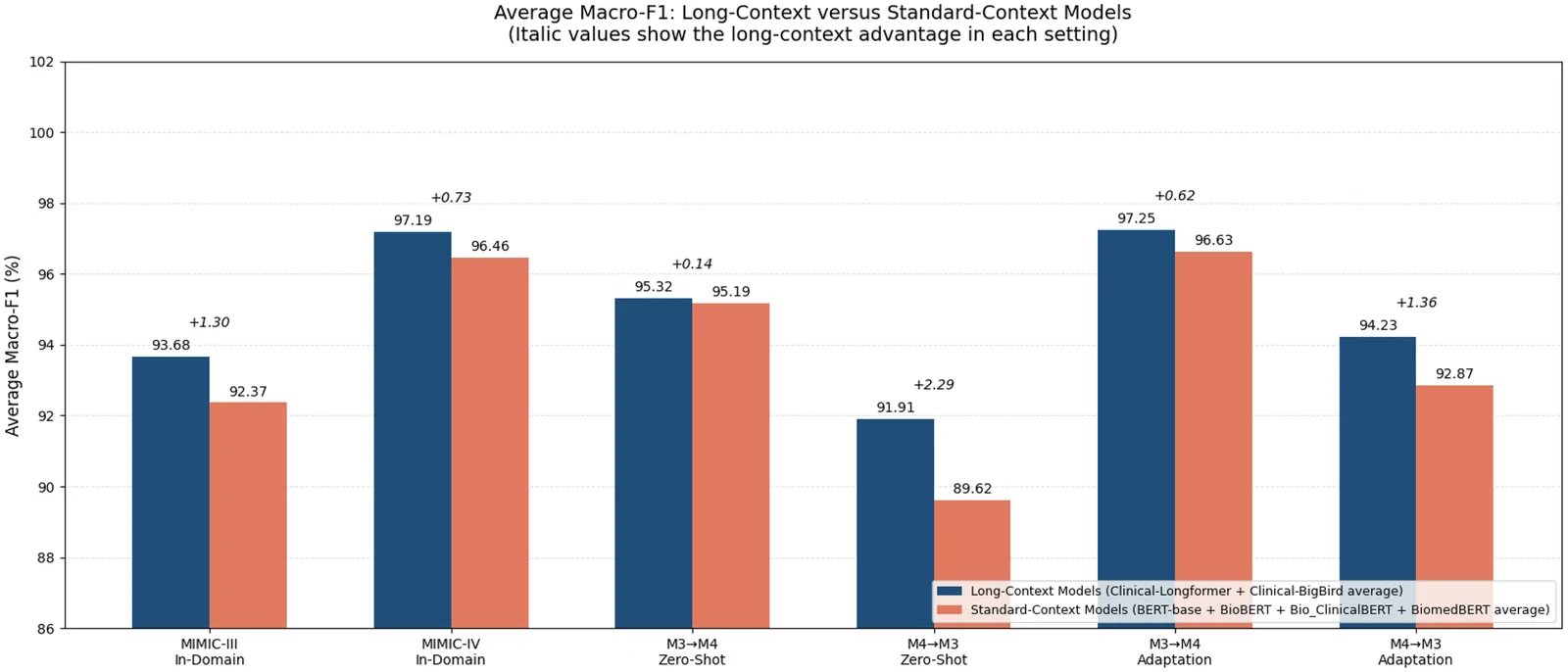
Average macro-F1 of long-context models and standard-context models across all experimental settings.

Figure 3 compares the average macro-F1 of the long-context group, consisting of Clinical-Longformer and Clinical-BigBird, with that of the standard-context group, consisting of BERT-base, BioBERT, Bio_ClinicalBERT, and BiomedBERT, across all six experimental settings. This comparison provides a setting-level summary of the contribution of broader context windows beyond model-specific variation.

The long-context group achieved a higher average macro-F1 in all six experimental settings. Clinical-BigBird achieved the highest individual macro-F1 in four settings, namely MIMIC-III in-domain, M3 → M4 zero-shot, M3 → M4 adaptation, and M4 → M3 adaptation. Clinical-Longformer achieved the highest individual performance in MIMIC-IV in-domain evaluation and M4 → M3 zero-shot transfer. In the M3 → M4 zero-shot setting, the long-context group achieved an average macro-F1 of 95.32%, compared with 95.19% for the standard-context group, corresponding to the smallest difference of 0.14 percentage points. The largest average advantage was observed in the M4 → M3 zero-shot setting, where the long-context group exceeded the standard-context group by 2.29 percentage points, followed by M4 → M3 adaptation with an advantage of 1.36 percentage points. Overall, these results indicate that the practical value of 4,096-token modeling is most evident under difficult inter-dataset transfer and target-domain adaptation conditions, while standard-context models remain competitive in less demanding transfer settings.

#### 4.5.1 Statistical significance analysis

Pairwise statistical significance testing was conducted using saved predictions from the four models included in the primary statistical comparison set, namely Clinical-Longformer, Clinical-BigBird, Bio_ClinicalBERT, and BiomedBERT, evaluated on the same subject-disjoint test instances. McNemar's test was used to compare model correctness patterns, and Holm–Bonferroni correction was applied across the 30 predefined primary comparisons to control the family-wise error rate at *α* = 0.05. Subject-level paired bootstrap resampling with 1,000 replicates was additionally used to estimate 95% confidence intervals and bootstrap *p*-values for the macro-F1 difference between the strongest long-context model and the strongest standard-context model in each experimental setting. BERT-base and BioBERT were included in the expanded predictive evaluation but were not included in the primary McNemar comparison family.

Table 4 reports six selected comparisons between the strongest long-context model and the strongest standard-context baseline in each setting. BERT-base and BioBERT are not shown because neither model was the strongest standard-context baseline in any of the six experimental settings.

**Table 4:** Statistical comparison between the strongest long-context and standard-context models in each experimental setting.

Experimental setting	Long-context model	Standard-context model	Macro-F1 difference (percentage points)	95% confidence interval	Bootstrap *p*-value	Holm-adjusted McNemar result
MIMIC-III in-domain	Clinical-BigBird	BiomedBERT	+0.80	0.19–1.40	0.008	Significant
MIMIC-IV in-domain	Clinical-Longformer	BiomedBERT	+0.59	0.15–1.11	0.004	Significant
M3 → M4 zero-shot	Clinical-BigBird	Bio_ClinicalBERT	+0.62	0.19–1.10	0.010	Significant
M4 → M3 zero-shot	Clinical-Longformer	BiomedBERT	+1.75	0.90–2.55	<0.001	Significant
M3 → M4 adaptation	Clinical-BigBird	BiomedBERT	+0.64	0.31–0.99	<0.001	Significant
M4 → M3 adaptation	Clinical-BigBird	BiomedBERT	+1.24	0.70–1.84	<0.001	Significant

The subject-level bootstrap confidence intervals remained above zero for all six selected comparisons, providing statistical support for the macro-F1 advantage of the strongest long-context model over the strongest standard-context baseline in each setting. The largest difference was observed in M4 → M3 zero-shot transfer, where Clinical-Longformer exceeded BiomedBERT by approximately 1.75 percentage points in macro-F1. This result supports the greater benefit of long-context modeling in the more difficult transfer direction.

However, the pairwise McNemar results showed that the advantage of one long-context architecture over another, or over every statistically tested standard-context comparator, was not uniform. In the M3 → M4 zero-shot setting, the differences between Clinical-Longformer and the two statistically tested standard-context baselines, Bio_ClinicalBERT and BiomedBERT, were not statistically significant after Holm correction. Similarly, the difference between Clinical-Longformer and Clinical-BigBird was not significant in either in-domain evaluation or in the M4 → M3 adaptation setting. These findings indicate that model superiority depends on the dataset and transfer condition rather than representing a uniform advantage across all comparisons.

#### 4.5.2 Computational efficiency analysis

Computational efficiency was evaluated using the final saved checkpoints of all six models on the MIMIC-IV test set. The benchmark measured model parameter count, mean inference latency, throughput, and peak allocated GPU memory. The measurements were conducted using 1,000 test instances with a batch size of one, following 50 warm-up iterations and three complete timed repetitions. CPU tokenization was excluded, while CPU-to-GPU tensor transfer and the model forward pass were included. The computational efficiency results for all six models are summarized in Table 5.

**Table 5:** Computational efficiency of the evaluated models.

Model	Maximum sequence length	Parameters (M)	Training time	Inference latency (ms/instance)	Throughput (instances/s)	Peak Memory (GB)
Clinical-Longformer	4,096	148.66	Approximately 8–9 h*	55.53 ± 5.13	18.01	0.88
Clinical-BigBird	4,096	128.06	Approximately 8–9 h*	12.10 ± 4.50	82.66	0.98
Bio_ClinicalBERT	512	108.31	Approximately 5–6 h*	7.45 ± 0.67	134.19	0.43
BiomedBERT	512	109.49	Approximately 5–6 h*	7.48 ± 0.61	133.64	0.44
BERT-base	512	109.49	Approximately 5–6 h*	7.23 ± 0.10	138.25	0.44
BioBERT	512	108.31	Approximately 5–6 h*	7.25 ± 0.04	138.01	0.43

Exact automatically logged wall-clock training runtimes were unavailable. The reported ranges are approximate durations directly observed by the authors during execution on the NVIDIA A100-SXM4–40GB GPU. For Clinical-Longformer and Clinical-Bigbird, the reported durations represent the complete two-stage fine-tuning procedure, including the 1,024-token and 4,096-token stages. For Bio_ClinicalBERT and BiomedBERT, the reported durations represent their complete single-stage 512-token fine-tuning runs. Equivalent observed training-duration records were not retained for BERT-base and BioBERT. However, their parameter counts, inference latency, throughput, and peak allocated inference GPU-memory values were directly measured using the same standardized benchmark protocol.

In addition to the directly measured inference metrics, approximate wall-clock training durations were reported based on the durations observed by the authors during model execution. Exact train_runtime values were not retained in the saved training logs. Therefore, the reported training-time ranges should be interpreted as approximate author-observed durations rather than precisely logged runtime measurements.

The four standard-context models provided the lowest inference latency and highest throughput. BERT-base achieved the lowest mean inference latency of 7.23 ms per instance and the highest throughput of 138.25 instances per second. BioBERT required 7.25 ms per instance and processed approximately 138.01 instances per second. Bio_ClinicalBERT required 7.45 ms per instance and processed approximately 134.19 instances per second, whereas BiomedBERT required 7.48 ms per instance and processed approximately 133.64 instances per second. The peak allocated inference GPU-memory usage was 0.44 GB for BERT-base, 0.43 GB for BioBERT, 0.43 GB for Bio_ClinicalBERT, and 0.44 GB for BiomedBERT.

Among the long-context models, Clinical-BigBird provided the stronger inference-efficiency balance. It required 12.10 ms per instance and achieved a throughput of 82.66 instances per second. Clinical-Longformer required 55.53 ms per instance and achieved a throughput of 18.01 instances per second. Clinical-BigBird used slightly more peak allocated inference GPU memory than Clinical-Longformer, with values of 0.98 GB and 0.88 GB, respectively. Thus, Clinical-BigBird was substantially faster than Clinical-Longformer, although it did not require less inference memory.

The approximate training-duration observations also indicate greater computational overhead for the long-context models. Clinical-Longformer and Clinical-BigBird each required approximately 8–9 h for the complete two-stage fine-tuning procedure, whereas Bio_ClinicalBERT and BiomedBERT each required approximately 5–6 h under the reported experimental configuration. Equivalent observed training-duration records were not retained for BERT-base and BioBERT; therefore, these two models were not included in the training-time comparison. However, these values were observed during execution rather than obtained from automatically stored runtime logs. They should therefore be interpreted as approximate ranges and not as precise stage-wise runtime benchmarks. Direct comparison should also account for the fact that the long-context models used a two-stage procedure with maximum lengths of 1,024 and 4,096 tokens, whereas the standard-context models were fine-tuned in a single stage at 512 tokens.

The inference benchmark followed the observed MIMIC-IV test-set sequence-length distribution, in which most instances were substantially shorter than the maximum 4,096-token context window. Therefore, these measurements represent inference behavior under the sampled test distribution and should not be interpreted as a worst-case full-length 4,096-token benchmark. Clinical-BigBird also automatically reverted from block-sparse attention to full attention for sufficiently short sequences. Its reported efficiency therefore reflects the actual attention behavior used for the sampled test instances.

Overall, the efficiency analysis demonstrates a clear trade-off between predictive performance and computational cost. The standard-context models achieved lower inference latency, higher throughput, and lower peak allocated inference GPU-memory usage than the long-context models. The available training-duration observations for Bio_ClinicalBERT and BiomedBERT were also shorter than those of the long-context models, although equivalent training-duration records were not retained for BERT-base and BioBERT. In contrast, the long-context models provided stronger predictive performance in the more difficult transfer settings but required greater computational overhead. Clinical-BigBird offered a more computationally efficient long-context alternative to Clinical-Longformer. The parameter count, inference latency, throughput, and peak allocated inference GPU-memory values for all six models were directly measured using the standardized benchmark, whereas the training durations are reported as approximate author-observed ranges where available because exact automatically logged runtime records were not retained.

### 4.6 Per-class F1 analysis

Table 6 presents the per-class F1 scores for all six models across all six evaluation settings. Class-level analysis is important because macro-F1 alone does not reveal which temporal relation categories drive the observed performance differences.

**Table 6:** Per-class F1 scores of all six models across all experimental settings.

Setting	Model	F1-BEFORE (%)	F1-AFTER (%)	F1-OVERLAP (%)	F1-NONE (%)
MIMIC-III in-domain	Clinical-Longformer	97.38	84.62	92.91	99.09
MIMIC-III in-domain	Clinical-BigBird	97.55	85.67	92.93	99.26
MIMIC-III in-domain	Bio_ClinicalBERT	97.13	83.91	91.69	97.20
MIMIC-III in-domain	BiomedBERT	97.28	84.57	92.42	97.92
MIMIC-III in-domain	BERT-base	97.09	82.39	91.47	97.10
MIMIC-III in-domain	BioBERT	97.04	82.34	91.15	97.28
MIMIC-IV in-domain	Clinical-Longformer	98.25	92.74	98.61	99.68
MIMIC-IV in-domain	Clinical-BigBird	98.03	91.83	98.75	99.64
MIMIC-IV in-domain	Bio_ClinicalBERT	97.47	90.38	98.30	99.05
MIMIC-IV in-domain	BiomedBERT	97.84	91.23	98.63	99.23
MIMIC-IV in-domain	BERT-base	97.51	91.30	98.21	98.96
MIMIC-IV in-domain	BioBERT	97.54	90.47	98.30	98.97
M3 → M4 zero-shot	Clinical-Longformer	96.45	84.10	98.02	99.60
M3 → M4 zero-shot	Clinical-BigBird	97.44	88.97	98.35	99.63
M3 → M4 zero-shot	Bio_ClinicalBERT	96.72	88.80	97.76	98.64
M3 → M4 zero-shot	BiomedBERT	96.66	88.26	98.07	98.79
M3 → M4 zero-shot	BERT-base	96.79	88.10	97.79	98.84
M3 → M4 zero-shot	BioBERT	95.96	85.89	97.42	98.46
M4 → M3 zero-shot	Clinical-Longformer	97.16	81.90	92.16	98.22
M4 → M3 zero-shot	Clinical-BigBird	96.69	79.09	92.40	97.60
M4 → M3 zero-shot	Bio_ClinicalBERT	95.72	78.10	91.80	92.80
M4 → M3 zero-shot	BiomedBERT	96.34	79.04	91.84	95.23
M4 → M3 zero-shot	BERT-base	95.74	78.16	91.46	94.26
M4 → M3 zero-shot	BioBERT	96.00	73.29	91.36	92.67
M3 → M4 adaptation	Clinical-Longformer	98.05	91.62	98.69	99.69
M3 → M4 adaptation	Clinical-BigBird	98.35	93.07	98.84	99.69
M3 → M4 adaptation	Bio_ClinicalBERT	97.44	91.85	98.21	99.07
M3 → M4 adaptation	BiomedBERT	97.88	92.04	98.38	99.08
M3 → M4 adaptation	BERT-base	97.78	91.62	98.36	99.18
M3 → M4 adaptation	BioBERT	97.35	90.59	98.23	99.04
M4 → M3 adaptation	Clinical-Longformer	97.66	86.49	92.99	99.30
M4 → M3 adaptation	Clinical-BigBird	97.78	87.02	93.37	99.19
M4 → M3 adaptation	Bio_ClinicalBERT	97.23	84.48	91.99	97.27
M4 → M3 adaptation	BiomedBERT	97.25	84.63	92.48	98.02
M4 → M3 adaptation	BERT-base	97.38	84.23	91.63	97.21
M4 → M3 adaptation	BioBERT	97.42	84.77	92.42	97.40

Across all six models and evaluation settings, NONE was generally the strongest and most stable class, while AFTER was consistently the most difficult class. BEFORE and OVERLAP also achieved strong performance, although their relative ordering varied across datasets and experimental settings. In particular, OVERLAP sometimes exceeded BEFORE in MIMIC-IV-based evaluations; therefore, a single fixed ordering between these two classes was not observed.

The NONE class often approached or exceeded 99% F1, particularly in the in-domain and adaptation settings. However, this result should be interpreted in light of the rule-based label construction: NONE indicates event pairs for which a deterministic temporal relation could not be assigned because one or both timestamps were unresolved. BEFORE and OVERLAP were also consistently strong, whereas AFTER remained substantially more difficult because it requires accurate direction-sensitive temporal ordering. Across the six models, AFTER F1 ranged from 73.29% for BioBERT under M4 → M3 zero-shot transfer to 93.07% for Clinical-BigBird under M3 → M4 adaptation.

**Figure 4:**
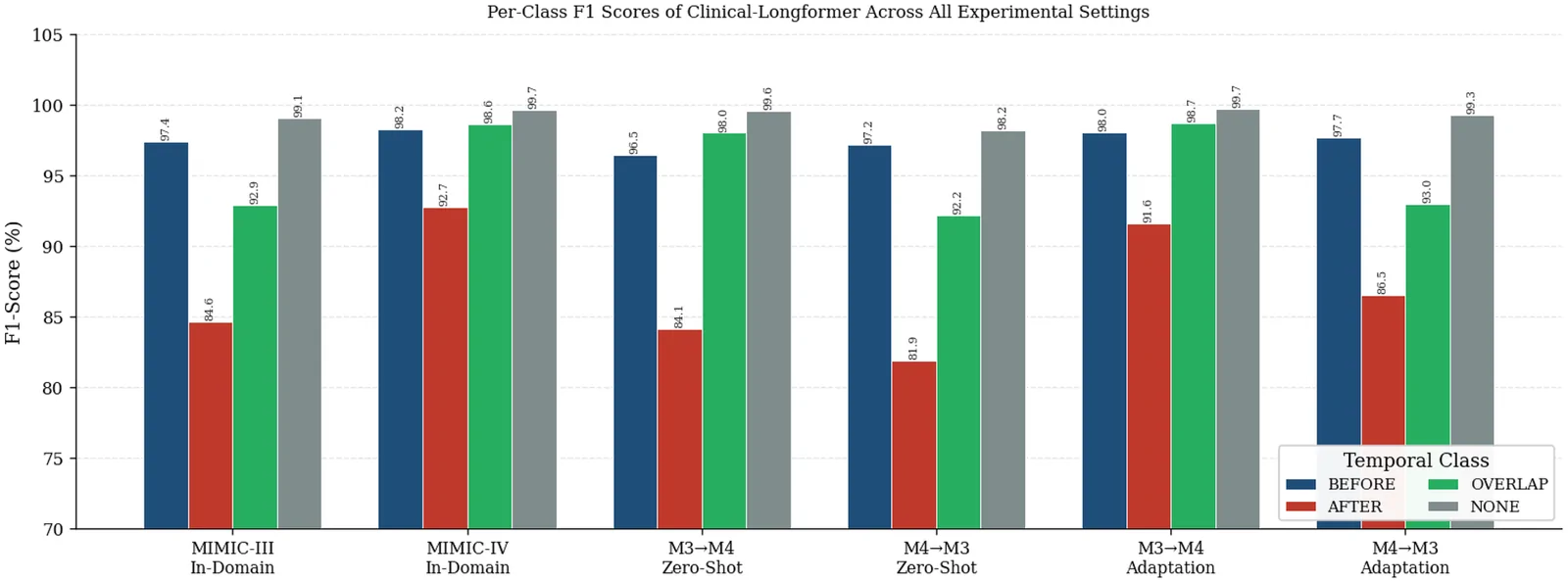
Per-class F1 scores of Clinical-Longformer across all six experimental settings.

**Figure 5:**
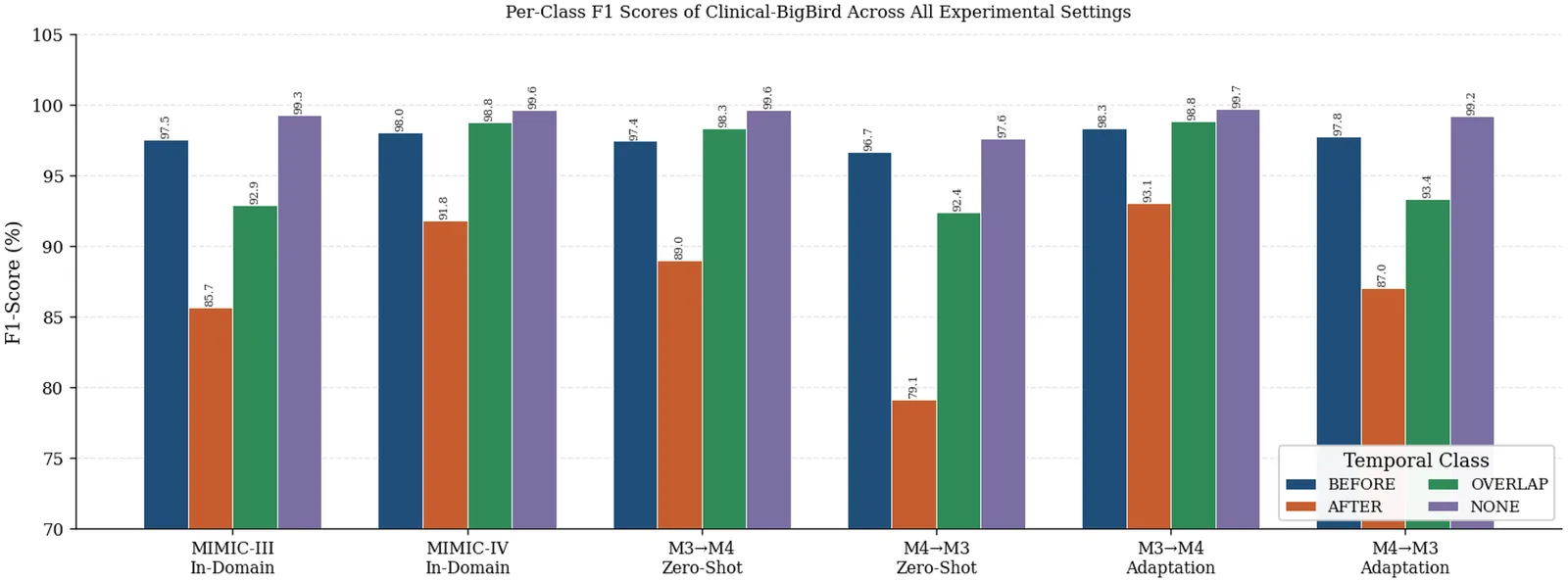
Per-class F1 scores of Clinical-BigBird across all six experimental settings.

The long-context models generally demonstrated stronger AFTER performance in the more difficult M4 → M3 transfer settings. Under M4 → M3 zero-shot transfer, Clinical-Longformer achieved the highest AFTER F1 of 81.90%, compared with 79.09% for Clinical-BigBird, 79.04% for BiomedBERT, 78.16% for BERT-base, 78.10% for Bio_ClinicalBERT, and 73.29% for BioBERT. After target-domain adaptation, Clinical-BigBird and Clinical-Longformer improved to 87.02% and 86.49%, respectively, remaining above all four standard-context baselines. This pattern suggests that broader contextual access may be particularly useful for direction-sensitive temporal decisions under more challenging dataset-transfer conditions. The per-class F1 behavior of Clinical-Longformer and Clinical-BigBird is further illustrated in Figures 4, 5, respectively.

### 4.7 Confusion matrix analysis

**Figure 6:**
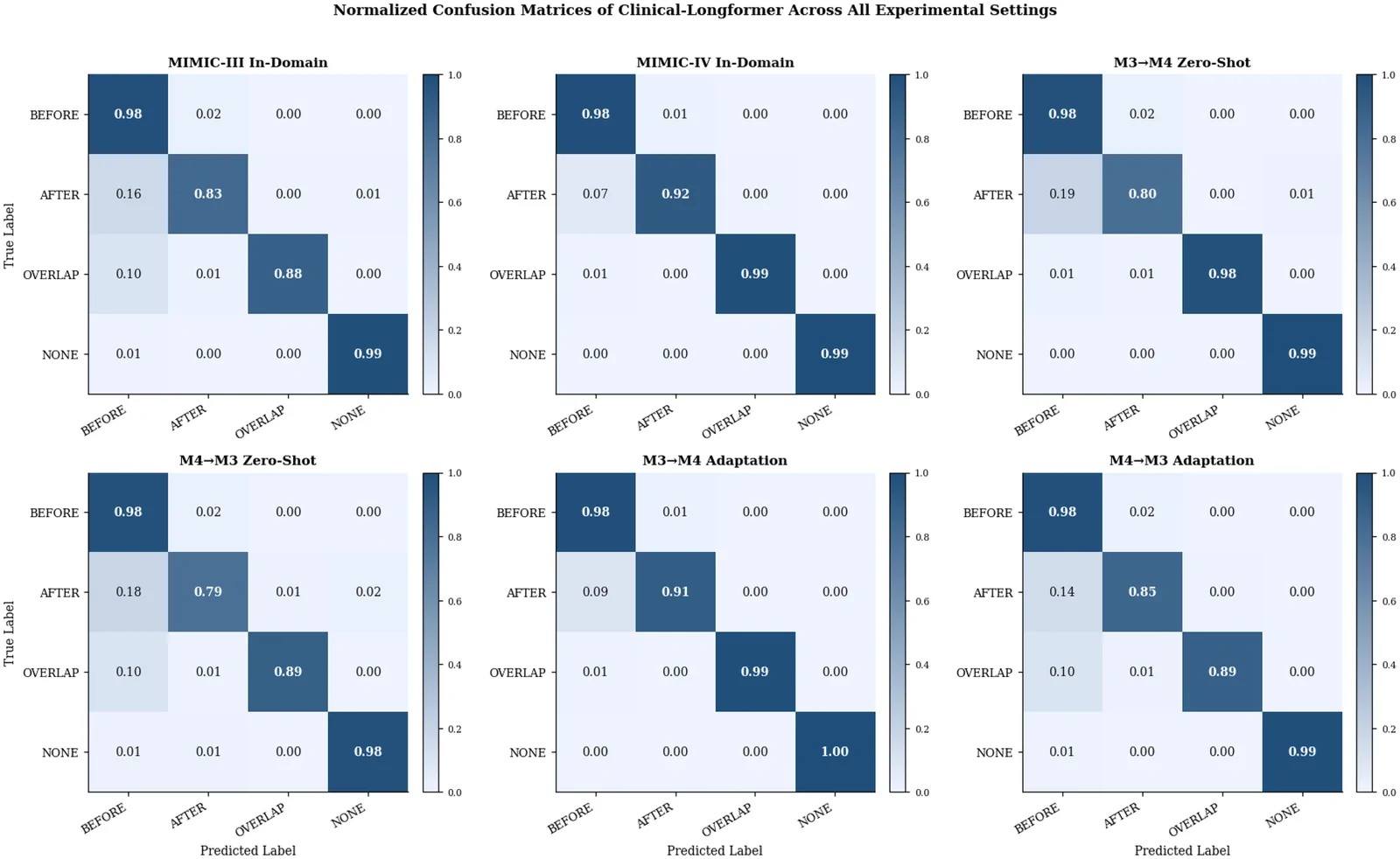
Normalized confusion matrices of Clinical-Longformer across all experimental settings.

**Figure 7:**
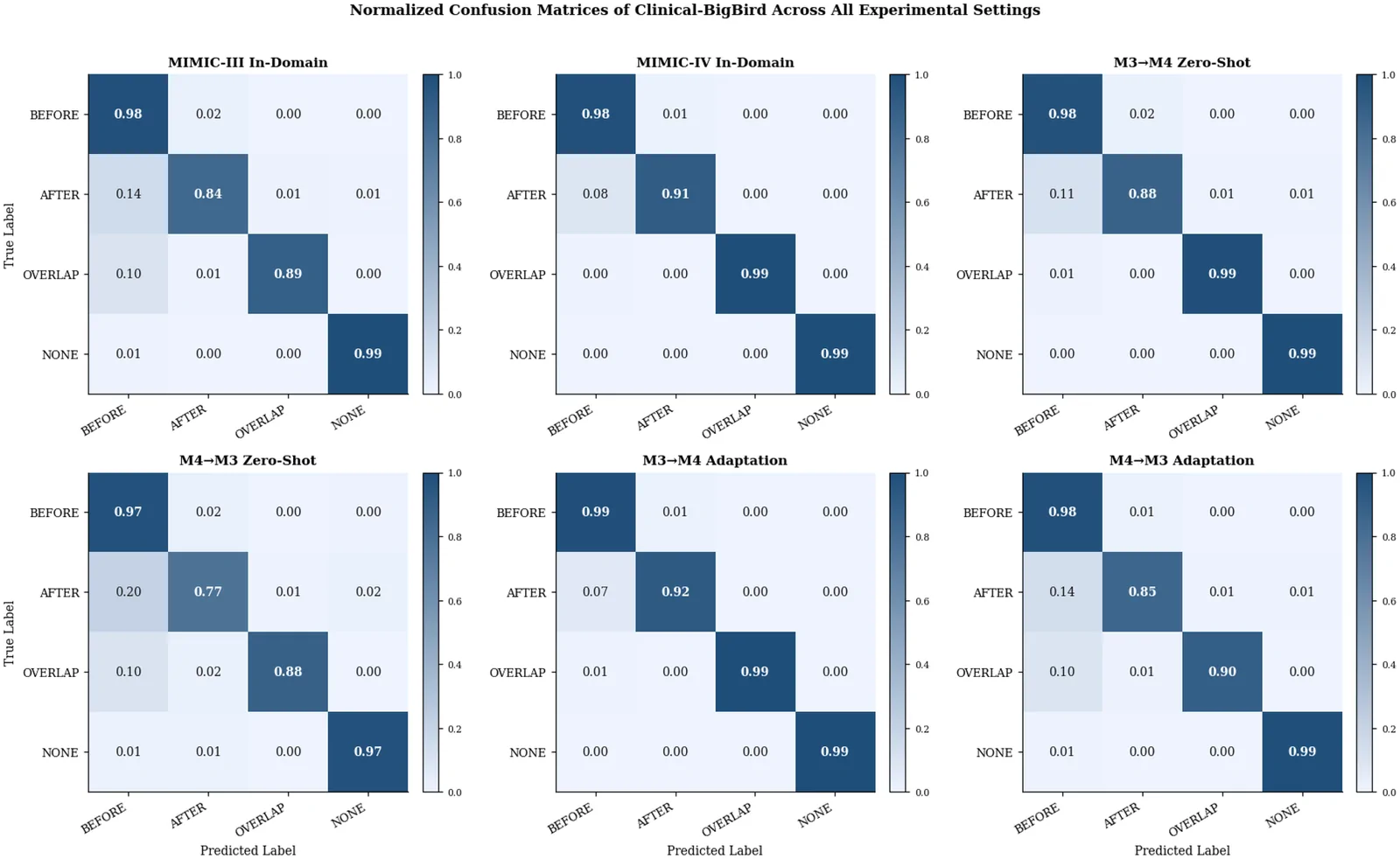
Normalized confusion matrices of Clinical-BigBird across all experimental settings.

Normalized confusion matrices were computed for Clinical-Longformer and Clinical-BigBird across all six evaluation settings to examine the internal structure of misclassification behavior. Figures 6,7 present these matrices for Clinical-Longformer and Clinical-BigBird, respectively.

For both models, the strongest diagonal values were observed for NONE and BEFORE across settings, in agreement with their high class-wise F1 scores. The dominant error pattern involved confusion between AFTER and BEFORE, which was most pronounced in the M4 → M3 zero-shot setting. A secondary but less severe confusion pattern between OVERLAP and BEFORE was also visible. Following target-domain adaptation, the confusion matrices showed clearer diagonal concentration, with the AFTER row exhibiting one of the most noticeable improvements. These observations suggest that a substantial portion of the transfer degradation is associated with corpus-level distributional mismatch rather than a general inability of the models to learn the rule-derived temporal ordering patterns.

#### 4.7.1 Qualitative error analysis

To complement the confusion-matrix analysis, a structured manual review was conducted on 120 misclassified instances sampled across the two long-context models and six experimental settings. The analysis showed that a large proportion of the reviewed errors were associated with limitations in the automatic preprocessing and silver-label generation pipeline rather than with the temporal-relation classifiers alone. Specifically, 72 cases (60.00%) were assigned to the primary category of possible silver-label noise, while 42 cases (35.00%) were assigned to abbreviation, formatting, or sentence-segmentation issues. Four cases (3.33%) involved ambiguous or missing temporal anchors, and one case each (0.83%) involved multiple competing temporal expressions and same-day or time-granularity ambiguity. The primary categories were mutually exclusive; therefore, the category counts summed to the complete set of 120 reviewed errors. The structured qualitative error-analysis results are summarized in Table 7.

**Table 7:** Structured qualitative analysis of 120 model misclassifications.

Primary error category	Number of instances	Percentage	Principal interpretation	Potential improvement
Possible silver-label noise	72	60.00%	The reference relation may have been affected by invalid candidate spans, incorrect datetime resolution, or errors in automatic relation assignment.	Improve event-span validation, temporal normalization, and silver-label auditing.
Abbreviation, formatting, or sentence-segmentation problems	42	35.00%	Numerical measurements, medication ratios, laboratory values, formatting artifacts, or incomplete spans were incorrectly treated as temporal candidates.	Improve clinical-text preprocessing, sentence segmentation, and numerical-expression filtering.
Ambiguous or missing temporal anchors	4	3.33%	The available context did not provide a sufficiently clear reference date or anchor for resolving the relation.	Strengthen admission-level and note-level temporal-anchor resolution.
Multiple competing temporal expressions	1	0.83%	Several nearby dates or temporal expressions made it difficult to identify the expression relevant to the event pair.	Apply context-aware temporal-expression selection and event–time linking.
Same-day or time-granularity ambiguity	1	0.83%	Calendar-date resolution could not distinguish events occurring at different times on the same date.	Use finer-grained hour- or minute-level timestamps when available.
Total	120	100.00%	—	—

Many reviewed cases contained numerical measurements, medication formulation ratios, laboratory values, hemodynamic readings, or incomplete spans affected by formatting and de-identification. Such expressions were incorrectly treated as temporal event candidates by the rule-based preprocessing pipeline. The concentration of reviewed errors in these upstream categories indicates that the classification models were generally more reliable when the marked spans represented valid and clearly anchored temporal events.

Together, possible silver-label noise and abbreviation, formatting, or sentence-segmentation problems accounted for 114 of the 120 reviewed errors, corresponding to 95.00% of the qualitative error sample. This finding indicates that most apparent model failures originated before temporal-relation classification, particularly during event-candidate detection, span construction, timestamp resolution, or automatic label assignment. Therefore, improvements to the upstream preprocessing and silver-label generation pipeline may produce greater performance gains than changes to the transformer classifier alone. However, because these cases were selected from model misclassifications, the 95.00% value describes only the reviewed error sample and does not estimate the prevalence of these problems across the complete datasets.

A smaller number of genuine temporal-reasoning errors were observed in instances containing closely positioned dates, reordered temporal evidence, or unclear temporal anchors. In one representative case, two medication-administration dates appeared close together and in a different narrative order across related medication instructions. The reference relation was AFTER, whereas the model predicted OVERLAP, suggesting that the proximity and reordering of the date expressions made the correct temporal direction more difficult to determine.

This representative case is consistent with the class-wise and confusion-matrix findings, in which direction-sensitive relations involving AFTER were more difficult than NONE and BEFORE. In particular, AFTER–BEFORE and AFTER–OVERLAP errors may occur when chronological order differs from narrative order, several dates are positioned close together, or both events share similar local context. These cases require the model to associate each marked event with the correct temporal expression before determining the direction of the relationship. Thus, even after invalid candidates and noisy labels are reduced, direction-sensitive temporal reasoning is likely to remain an important source of residual error.

Overall, the qualitative findings indicate that the residual errors arise from a combination of genuine direction-sensitive temporal reasoning difficulties and noise inherited from the automatic candidate-extraction and silver-labeling pipeline. These results suggest that further performance improvements may be achieved by refining event-span validation, temporal-expression normalization, and silver-label generation. More specifically, the findings support incorporating numerical-expression filters, stricter temporal-candidate validation, improved event–time linking, and finer-grained timestamp resolution where hour- or minute-level information is available. Because the reviewed cases were sampled specifically from model misclassifications, the reported proportions characterize only the examined error sample and should not be interpreted as the overall label-noise rate of the complete datasets. This qualitative error analysis was conducted separately from the physician review described in Section 4.7.2, which assessed the correctness of the original silver labels rather than the sources of model misclassification.

#### 4.7.2 Physician assessment of silver-label reliability

In addition to the qualitative review of model misclassifications, a separate physician review was conducted to assess the reliability of the rule-derived silver labels. The physician reviewed 200 event-pair instances and evaluated whether the original silver temporal-relation label was correct or incorrect.

Of the 200 reviewed instances, 160 labels were judged correct and 40 labels were judged incorrect. This corresponds to a physician-agreement rate of 80.0% and an observed silver-label disagreement rate of 20.0%. The approximate 95% Wilson confidence interval was 73.9%–85.0% for physician agreement and 15.0%–26.1% for disagreement.

These findings indicate that most labels in the physician-reviewed sample were consistent with the physician's assessment. Nevertheless, the observed 20.0% disagreement demonstrates that the automatic rule-based labeling procedure introduced a measurable amount of annotation noise. Therefore, the reported model scores should be interpreted as performance against rule-derived supervision rather than as direct performance against a fully expert-annotated gold standard.

Because the physician review was limited to 200 event-pair instances, the observed disagreement rate represents the reviewed sample and should not be interpreted as an exact population-wide noise estimate for the complete MIMIC-derived datasets. The physician-assessment results are summarized in Table 8.

**Table 8:** Physician assessment of rule-derived silver labels.

Physician-review outcome	Number of instances	Percentage	95% Wilson CI
Correct silver labels	160	80.0%	73.9%–85.0%
Incorrect silver labels	40	20.0%	15.0%–26.1%
Total reviewed	200	100.0%	—

The disagreement rate represents physician-identified label noise within the reviewed sample and is not treated as an exact population-wide noise estimate.

### 4.8 Ablation study: effect of the second-stage 4,096-token continuation

To examine the effect of the second-stage continuation, the Stage 1-only checkpoints were compared with the final checkpoints obtained after one additional epoch at a maximum sequence length of 4,096 tokens. This comparison evaluates whether the complete two-stage procedure improves performance over Stage 1 training alone. However, because Stage 2 simultaneously adds further optimization and increases the maximum context length, the experiment does not fully isolate the benefit attributable specifically to the 4,096-token context window.

Specifically, two configurations were evaluated for each model. In the Stage 1-only configuration, the model was fine-tuned at 1,024 tokens for 2 epochs with validation-based model selection and early stopping, and Stage 2 was omitted entirely. In the full two-stage configuration, the best Stage 1 checkpoint was further fine-tuned for 1 epoch at 4,096 tokens with a reduced learning rate. The ablation was evaluated on the in-domain test sets for both MIMIC-III and MIMIC-IV using Clinical-Longformer and Clinical-BigBird, with the same leakage-free subject-level splits and evaluation protocol as elsewhere in the study. The complete accuracy and macro-F1 results for both configurations, together with the per-model gain from Stage 2, are reported in Table 9.

**Table 9:** Ablation study results for stage 1 only (1,024 tokens) and the full two-stage pipeline (4,096 tokens).

Model	Dataset	Stage 1 only accuracy (%)	Stage 1 only macro-F1 (%)	Stage 1 + 2 accuracy (%)	Stage 1 + 2 macro-F1 (%)	Accuracy gain	Macro-F1 gain
Clinical-Longformer	MIMIC-III	95.30	91.80	96.45	93.50	+1.14%	+1.70%
Clinical-Longformer	MIMIC-IV	98.26	96.87	98.55	97.32	+0.29%	+0.46%
Clinical-BigBird	MIMIC-III	95.78	92.52	96.69	93.85	+0.90%	+1.34%
Clinical-BigBird	MIMIC-IV	97.68	95.73	98.41	97.06	+0.73%	+1.33%

Across all reported model-dataset combinations, removing Stage 2 reduced both accuracy and macro-F1. The largest macro-F1 reduction was observed with Clinical-Longformer on MIMIC-III, where performance decreased from 93.50% to 91.80%, a drop of 1.70 percentage points. Clinical-BigBird on MIMIC-III also showed a clear decrease, from 93.85% to 92.52%. In MIMIC-IV, the magnitude of the reduction was smaller with Clinical-Longformer but remained consistent, whereas Clinical-BigBird again showed a notable decrease when Stage 2 was removed.

Paired McNemar tests were conducted to determine whether the improvements produced by Stage 2 were statistically significant. Holm correction was applied across the four ablation comparisons to control for multiple testing. All four improvements remained statistically significant after correction. The adjusted *p*-values were 2.11 × 10^−19^ for Clinical-Longformer on MIMIC-III, 4.37 × 10^−9^ for Clinical-Longformer on MIMIC-IV, 3.29 × 10^−16^ for Clinical-BigBird on MIMIC-III, and 1.85 × 10^−21^ for Clinical-BigBird on MIMIC-IV. These results confirm that the improvements obtained after Stage 2 were not limited to numerical variations in the aggregate performance measures.

Overall, the ablation results indicate that the 4,096-token continuation provides statistically significant performance gains beyond 1,024-token Stage 1 training alone. Although the magnitude of improvement varies by model and corpus, the direction of the effect is consistent across all evaluated configurations. These findings support the inclusion of Stage 2 as a meaningful component of the proposed long-context training framework.

### 4.9 Novelty and comparison with existing work

The novelty of the present study is methodological and empirical rather than architectural The study does not introduce a new transformer architecture, as all six evaluated backbones, Clinical-Longformer, Clinical-BigBird, BERT-base, BioBERT, Bio_ClinicalBERT, and BiomedBERT, are existing pretrained models. Instead, the contribution lies in a unified robustness-oriented experimental framework that combines large-scale rule-derived temporal relation datasets from MIMIC-III and MIMIC-IV, a four-class formulation that includes the NONE category, leakage-free subject-level data partitioning, a comparison of long-context and standard-context clinical encoders, and evaluation under in-domain, bidirectional zero-shot, and adaptation-based transfer settings.

Direct numerical comparison with prior clinical temporal relation extraction systems is not appropriate because existing studies use substantially different datasets, label spaces, annotation strategies, task formulations, and evaluation protocols. Much of the prior literature has been evaluated on benchmark corpora such as i2b2 2,012 and THYME, which generally contain smaller manually annotated datasets and different temporal-relation formulations. In contrast, the present study evaluates large-scale prepared event-pair datasets derived from MIMIC-III and MIMIC-IV using a four-class formulation that includes an additional NONE category. Accordingly, the comparison with previous studies is presented qualitatively rather than as a direct score-based benchmark.

Recent clinical temporal-relation research has increasingly moved beyond conventional BERT-based pair classification. Recent studies have explored text and knowledge-graph integration, graph-based transformer architectures, zero-shot large language models, and LLM-based clinical information extraction. The ChemoTimelines shared tasks further expanded the scope of temporal information extraction by focusing on event–time relations and the construction of clinically meaningful treatment timelines. These developments demonstrate increasing interest in document-level temporal reasoning, graph-based dependency modelling, and generative clinical NLP. However, the reviewed studies were mainly evaluated on i2b2, THYME, pediatric, or oncology-specific datasets and did not examine bidirectional transfer between MIMIC-III and MIMIC-IV under the unified evaluation protocol adopted in the present study.

As shown in Table 10, prior work has predominantly focused on smaller benchmark corpora, manually annotated temporal relations, and within-corpus evaluation. In contrast, the present study differs in three principal respects. First, it evaluates temporal relation extraction on both MIMIC-III and MIMIC-IV rather than on a single benchmark corpus. Second, it adopts a four-class formulation that includes the NONE category, which represents event pairs for which the timestamp-resolution pipeline could not assign a deterministic temporal relation. Third, it explicitly evaluates robustness using in-domain testing, bidirectional zero-shot inter-dataset transfer, and adaptation-based transfer within a unified experimental framework. These characteristics position the present study as a robustness-oriented evaluation of long-context clinical transformers across two major EHR corpora.

**Table 10:** Qualitative comparison between the present study and prior clinical temporal relation extraction works.

Study	Year	Dataset	Classes	Model architecture	Cross-dataset evaluation	Long-context encoding
Zhou et al.	2021	i2b2 2012	3 (B/A/O)	BERT + global inference	No	No
Cheng & Weiss	2023	i2b2 2012	3 (B/A/O)	BERT + typed markers	No	No
Knez et al.	2024	i2b2, THYME	3 (B/A/O)	BERT + knowledge graph	No	No
Kougia et al.	2024	i2b2 2012	3 (B/A/O)	Zero-shot LLMs	No	No
Andrew et al.	2025	Pediatric EHR	Binary	LLM-based extraction	No	No
Chaturvedi et al.	2025	i2b2 2012	3 (B/A/O)	Span-based graph transformer	No	Partial
Present study	2026	MIMIC-III, MIMIC-IV	4 (B/A/O/N)	Clinical-Longformer/Clinical-BigBird	Yes	Yes

B = BEFORE, A = AFTER, O = OVERLAP, N = NONE.

The predominant benchmark in earlier clinical temporal relation extraction research has been the i2b2 2012 corpus, although later studies have also considered THYME, ChemoTimelines, and other clinical datasets. Existing research has investigated BERT-based classifiers, typed-marker approaches, bimodal architectures combining textual and structured knowledge, graph-based transformer frameworks, zero-shot large language models, and LLM-based clinical information-extraction approaches. However, these studies have generally focused on manually annotated benchmark corpora and within-corpus evaluation rather than large-scale silver-labeled event-pair datasets derived from MIMIC-III and MIMIC-IV. Moreover, the reviewed studies did not evaluate robustness using the same unified combination of in-domain evaluation, bidirectional zero-shot inter-dataset transfer, and adaptation-based transfer across MIMIC-III and MIMIC-IV.

The present study also differs from prior work in ways that prevent direct numerical comparison. First, the evaluation is conducted on large-scale prepared event-pair datasets comprising 159,542 MIMIC-III instances and 209,482 MIMIC-IV instances. These instances were generated by applying an automatic rule-based temporal reasoning pipeline to discharge summaries rather than by using manually annotated gold labels such as those available in i2b2. The use of rule-derived silver labels enables evaluation at a scale that would be difficult to achieve through expert annotation alone. Second, the four-class label space, including the NONE category, introduces an additional classification condition that is absent from conventional three-class benchmark formulations. Third, the experimental design includes bidirectional zero-shot and adaptation-based transfer evaluation between two large EHR corpora, allowing model robustness to be examined under dataset variation.

To the best of our knowledge, based on the literature reviewed for this study, no previously published work has evaluated the same large-scale, four-class, MIMIC-derived event-pair formulation under a combined protocol of in-domain evaluation, bidirectional zero-shot transfer, and adaptation-based transfer. The contribution of the present study is therefore not a new transformer architecture, but a distinct robustness-oriented framework for evaluating long-context and standard-context clinical encoders across two major EHR corpora. This contribution complements recent graph-based, zero-shot, and large-language-model-based approaches by focusing specifically on contextual length, dataset shift, and bidirectional transfer robustness.

### 4.10 Discussion

The results show that long-context clinical transformers provide setting-dependent advantages for rule-derived temporal relation classification. Standard-context baselines remained competitive in matched in-domain evaluation and the easier MIMIC-III-to-MIMIC-IV zero-shot direction, whereas long-context models showed clearer benefits in the more difficult MIMIC-IV-to-MIMIC-III transfer direction and in both adaptation settings. These findings suggest that the two-stage procedure improves robustness under differences in documentation style, class distribution, and corpus structure. However, because Stage 2 introduces both additional optimization and a larger context window, its gains cannot be attributed exclusively to 4,096-token modeling.

The additional BERT-base and BioBERT experiments strengthened the generalization analysis. Both models reproduced the directional transfer asymmetry observed with the original models and improved after target-domain adaptation. Thus, the main transfer and adaptation patterns were consistent across general-domain, biomedical-domain, clinical-domain, and long-context clinical pretrained encoders.

Class-wise results showed that NONE and BEFORE were generally easier than AFTER. The difficulty of AFTER reflects the need to identify direction-sensitive temporal ordering rather than unresolved or same-date relations. The stronger AFTER performance of the long-context models, particularly in MIMIC-IV-to-MIMIC-III zero-shot transfer, suggests that broader contextual access may support difficult temporal decisions. Nevertheless, these findings represent performance against rule-derived silver labels rather than expert-annotated clinical reasoning.

The physician review confirmed 160 of 200 silver labels, corresponding to 80.0% agreement and 20.0% disagreement. This result indicates that the rule-derived supervision was useful but contained measurable annotation noise. Such noise may either underestimate performance by treating clinically correct predictions as errors or inflate performance when models learn pipeline-specific artifacts. Because the review involved only 200 instances and one physician, the observed disagreement should not be interpreted as a population-wide estimate.

Target-domain adaptation reduced cross-corpus degradation across all models, indicating that limited refinement can help models adjust to corpus-specific temporal expressions and label distributions. Clinical-BigBird showed the strongest adaptation performance in both directions, whereas Clinical-Longformer performed best in MIMIC-IV in-domain evaluation and MIMIC-IV-to-MIMIC-III zero-shot transfer. These results indicate complementary strengths between the two long-context models.

The ablation study further supported the value of Stage 2. Removing the 4,096-token continuation reduced performance for both long-context models on both corpora, and all four Stage 1 vs. Stage 1 + 2 comparisons remained statistically significant after Holm correction. However, because Stage 2 changes both training duration and maximum context length, the ablation does not isolate the independent effect of the extended context window. The findings therefore support progressive 1,024-to-4,096-token continuation as a practical training strategy rather than proving that context length alone caused the gains.

The statistical analysis also supported setting-dependent rather than universal superiority. Subject-level bootstrap confidence intervals remained above zero for the strongest long-context vs. standard-context comparison in all six settings, with the largest macro-F1 difference occurring in MIMIC-IV-to-MIMIC-III zero-shot transfer. However, Clinical-Longformer did not significantly differ from Bio_ClinicalBERT or BiomedBERT in MIMIC-III-to-MIMIC-IV zero-shot transfer, and the two long-context models did not differ significantly in either in-domain setting or in MIMIC-IV-to-MIMIC-III adaptation.

The qualitative review showed that many apparent model errors originated from upstream processing. Of the 120 reviewed misclassifications, 72 were associated with possible silver-label noise and 42 with abbreviation, formatting, or sentence-segmentation problems. The remaining cases involved ambiguous temporal anchors, competing temporal expressions, or time-granularity issues. These findings suggest that improved candidate validation, temporal normalization, and silver-label generation may yield substantial gains. Because the cases were selected from model misclassifications, their proportions describe only the reviewed error sample.

The efficiency analysis revealed a clear trade-off between predictive performance and computational cost. Standard-context models achieved lower inference latency, higher throughput, and lower inference-memory usage. Clinical-BigBird provided the more efficient long-context alternative, whereas Clinical-Longformer required substantially greater inference time. The reported training durations are approximate because exact runtime logs were unavailable, and the memory values represent inference rather than peak training usage. In addition, the benchmark followed the observed MIMIC-IV test-set length distribution and should not be interpreted as a worst-case 4,096-token evaluation.

Several limitations should be acknowledged. First, the experimental design does not separate the effect of additional Stage 2 optimization from that of increased context length. Second, both datasets originate from the same hospital system, so the evaluation represents inter-version rather than cross-institution transfer. Third, not all recently developed extended-context biomedical encoders were evaluated. Fourth, each configuration was trained once, and the significance tests therefore do not measure variability across independent runs. Fifth, exact training runtimes and peak training-memory measurements were not retained, and equivalent training-duration observations were unavailable for BERT-base and BioBERT. Sixth, the efficiency benchmark reflects the observed test distribution rather than maximum-length inputs. Finally, the labels were generated automatically, the physician audit involved only 200 instances and one reviewer, and the qualitative analysis examined 120 selected misclassifications. Therefore, neither review provides a population-level estimate of label noise or error prevalence.

## 5 Conclusion

This study presented a long-context framework for rule-derived clinical temporal relation extraction from prepared event-pair instances derived from MIMIC-III and MIMIC-IV. The task was formulated as a silver-label four-class classification problem with labels BEFORE, AFTER, OVERLAP, and NONE, where NONE indicates event pairs for which the timestamp-resolution pipeline could not assign a deterministic temporal relation. The framework was evaluated in in-domain, zero-shot, and adaptation-based inter-dataset settings. Across these experiments, all six models achieved strong in-domain performance, while clearer differences emerged under transfer-oriented evaluation. Clinical-BigBird produced the strongest results on MIMIC-III in-domain evaluation and in both adaptation settings, whereas Clinical-Longformer achieved the best performance on MIMIC-IV in-domain evaluation and in the more difficult MIMIC-IV to MIMIC-III zero-shot setting. The expanded six-model evaluation further showed that BERT-base and BioBERT reproduced the same directional zero-shot transfer asymmetry and benefited from target-domain adaptation, supporting the consistency of the generalization findings across general-domain, biomedical, and clinical pretrained model families.

The findings indicate that the benefit of long-context modeling is most evident under conditions of high contextual complexity and dataset shift, rather than in a uniform manner across all settings. Although the 512-token baseline models remained competitive in some matched and less challenging transfer conditions, the long-context models showed stronger overall robustness, particularly in harder transfer scenarios and on the direction-sensitive AFTER class. In addition, the ablation study showed that the complete two-stage 1,024-to-4,096-token continuation procedure produced consistent gains across both long-context architectures and both corpora. The statistical analysis supported the differences between the strongest long-context and standard-context models in the selected setting-level comparisons, while also showing that not every pairwise model difference was statistically significant.

A separate physician review of 200 rule-derived event-pair labels confirmed 160 labels as correct, corresponding to a physician-agreement rate of 80.0%, while 40 labels were judged incorrect, corresponding to an observed disagreement rate of 20.0%. This finding provides a direct estimate of silver-label reliability within the reviewed sample and highlights the need to interpret model performance in the context of imperfect rule-derived supervision.

The computational analysis further showed that these predictive benefits involve additional measured inference costs. The standard-context models were faster and required less inference GPU memory, whereas Clinical-BigBird provided a more efficient long-context alternative to Clinical-Longformer. The qualitative review also indicated that many residual errors were associated with limitations in candidate extraction, preprocessing, and silver-label generation rather than temporal-relation classification alone.

Overall, these results suggest that long-context clinical transformers provide a practical approach for robust rule-derived temporal relation classification from EHR narratives, especially when temporal evidence is distributed across extended event-pair context windows and when models must generalize across corpus variation. However, the findings should be interpreted within the context of silver-label supervision, because the temporal relation labels were generated using a deterministic rule-based timestamp-resolution pipeline rather than manual expert annotation.

### 5.1 Future work

Several directions remain for future investigation. First, the proposed framework should be evaluated on more diverse clinical corpora from different institutions to assess its generalizability beyond the MIMIC setting. Second, future studies should validate the observed performance differences through repeated training runs and evaluation on independent external clinical datasets. Third, future work may compare the present two-stage continuation strategy against direct single-stage long-context training to better isolate the contribution of progressive context extension. Fourth, future studies should examine the framework on manually annotated temporal relation corpora to further assess whether the observed improvements transfer from silver-label supervision to expert-annotated clinical temporal reasoning.

Future work should extend the present physician audit to a larger and more representatively sampled subset of the complete datasets. Review by at least two clinical or temporal-annotation experts would permit the calculation of inter-reviewer agreement and provide a more precise estimate of silver-label reliability. Future improvements should additionally refine temporal candidate extraction, span validation, datetime normalization, and rule-based label generation to reduce errors caused by numerical values, formatting irregularities, incomplete spans, and ambiguous temporal anchors. Exact stage-wise training times and peak training-memory measurements should also be retained in future experiments to support more detailed computational comparisons. Finally, exploring newer extended-context biomedical encoders and more advanced adaptation strategies may further improve robustness, especially for difficult temporal relation types such as AFTER.

## Data Availability

Publicly available datasets were analyzed in this study. This data can be found here: The existing datasets analyzed in this study are available through PhysioNet under credentialed access requirements:MIMIC-III Clinical Database v1.4, PhysioNet: https://physionet.org/content/mimiciii/1.4/MIMIC-IV Clinical Database, PhysioNet: https://physionet.org/content/mimiciv/ The derived event-pair datasets generated during the current study are not publicly redistributed because of PhysioNet data-use restrictions. Further inquiries should be directed to the corresponding author/s.

## References

[1] Johnson AEW Bulgarelli L Shen L Gayles A Shammout A Horng S. MIMIC-IV, a freely accessible electronic health record dataset. Sci Data. (2023) 10(1):1–9. 10.1038/s41597-022-01899-x 36596836 PMC9810617

[2] Wu H Wang M Wu J Francis F Chang Y-H Shavick A. A survey on clinical natural language processing in the United Kingdom from 2007 to 2022. npj Digit Med. (2022) 5:186. 10.1038/s41746-022-00730-6 36544046 PMC9770568

[3] Gumiel YB Silva e Oliveira LE Claveau V Grabar N Paraiso EC Moro C. Temporal relation extraction in clinical texts. ACM Comput Surv (2022) 54(7):1–36. 10.1145/3462475

[4] Su X Howard P Bethard S. Transformer-based temporal information extraction and application: a review. In: Proceedings of the 2025 Conference on Empirical Methods in Natural Language Processing. Suzhou: Association for Computational Linguistics (2025). p. 28822–41. 10.18653/v1/2025.emnlp-main.1467

[5] Knez T Žitnik S. Multimodal learning for temporal relation extraction in clinical texts. J Am Med Informatics Assoc. (2024) 31(6):1380–7. 10.1093/jamia/ocae059 PMC11105141 38531680

[6] Andrew JJ Potier J Garcelon N Burgun A Vincent M. Using large language models for temporal relation extraction from pediatric clinical reports. JAMIA Open. (2025) 8(6):ooaf121. 10.1093/jamiaopen/ooaf121 41281245 PMC12640238

[7] Chaturvedi R Baghershahi P Medya S Di B. Temporal relation extraction in clinical texts : a span-based graph transformer approach. In: Proc. 63rd Annu. Meet. Assoc. Comput. Linguist.) (2025), 1, 25765–88

[8] Kougia V Sedova A Stephan A Zaporojets K Roth B. Analysing zero-shot temporal relation extraction on clinical notes using temporal consistency. In: Proceedings of the 23rd Workshop on Biomedical Natural Language Processing. Bangkok: Association for Computational Linguistics (2024). p. 72–84. 10.18653/v1/2024.bionlp-1.6

[9] Li Y Wehbe RM Ahmad FS Wang H Luo Y. A comparative study of pretrained language models for long clinical text. J Am Med Informatics Assoc. (2023) 30(2):340–7. 10.1093/jamia/ocac225 PMC9846675 36451266

[10] Li Y Wehbe RM Ahmad FS Wang H Luo Y. Clinical-longformer and clinical-BigBird: transformers for long clinical sequences. (2022). Available online at: https://arxiv.org/abs/2201.11838 (Accessed August 12, 2026).

[11] Peng C Yang X Yu Z Bian J Hogan WR Wu Y. Clinical concept and relation extraction using prompt-based machine Reading comprehension. J Am Med Informatics Assoc. (2023) 30(9):1486–93. 10.1093/jamia/ocad107 PMC10436141 37316988

[12] Builtjes L Bosma J Prokop M Van Ginneken B Hering A. Leveraging open-source large language models for clinical information extraction in resource-constrained settings. JAMIA Open. (2025) 8(5):ooaf109. 10.1093/jamiaopen/ooaf109 41041625 PMC12488231

[13] Yao J Hochheiser H Yoon WJ Goldner E Savova G. Overview of the 2024 shared task on chemotherapy treatment timeline extraction. In: Clin. 2024–6th Work. Clin. Nat. Lang. Process. Proc. Work.) (2024), 557–69. 10.18653/v1/2024.clinicalnlp-1.53

[14] Yao J Hochheiser H Yoon WJ Goldner E Savova G. Overview of the 2025 shared task on chemotherapy treatment timeline extraction. In: Proceedings Ofthe 7th Clinical Natural Language Processing Workshop) (2025), 1–10

[15] Zhao Z Vydiswaran VGV. Team NLP4Health at ChemoTimelines 2025: finetuning large language models for temporal relation extractions from clinical notes. In: Proc. 7th Clin. Nat. Lang. Process. Work. (2025), 22–9. Available online at: https://aclanthology.org/2025.clinicalnlp-1.4/ (Accessed August 8, 2026).

[16] Olex AL McInnes BT. Review of temporal reasoning in the clinical domain for timeline extraction: where we are and where we need to be. J Biomed Inform (2021) 118:103784. 10.1016/j.jbi.2021.103784 33862232

[17] Wang L Lu Q Li R Fu S Liu H. Wonder at chemotimelines 2024: medTimeline: an End-to-End NLP system for timeline extraction from clinical narratives. In: Proceedings of the 6th Clinical Natural Language Processing Workshop (Stroudsburg, PA, USA: Association for Computational Linguistics) (2024), 483–7. 10.18653/v1/2024.clinicalnlp-1.48

[18] Cui H Unell A Chen B Fries JA Alsentzer E Koyejo S. TIMER: temporal instruction modeling and evaluation for longitudinal clinical records. npj Digit Med (2025) 8(1):577. 10.1038/s41746-025-01965-9 41006898 PMC12475073

[19] Yoon WJ Chen S Gao Y Zhao Z Dligach D Bitterman DS. LCD benchmark: long clinical document benchmark on mortality prediction for language models. medRxiv. (2024) 27:2024.03.26.24304920. 10.1101/2024.03.26.24304920 PMC11756648 39602813

[20] Rodrigues T Teixeira Lopes C. Harnessing large language models for clinical information extraction: a systematic literature review. ACM Trans Comput Healthc. (2025) 6(4):1–35. 10.1145/3744660

[21] Farhadi A Zamanifar A Bahrpeyma F. Explainable AI (XAI): making LLM decisions transparent and trustworthy. In: Zamanifar A Faezipour M Gharehchopogh FS Farhadi A, editors. Applications of Large Language Models (LLM) in Healthcare Systems. Boca Raton: Chapman and Hall/CRC (2025). p. 179–208. 10.1201/9781003665083-7

[22] Zamanifar A Taherkordi A Farhadi A, editors. Explainable Large Language Models in Healthcare Applications. Bio-IT and AI. Cham: Springer Nature Switzerland (2026). 10.1007/978-3-032-15088-2

[23] Vasilakes J Georgiadis P Nguyen NTH Miwa M Ananiadou S. Contextualized medication event extraction with levitated markers. J Biomed Inform (2023) 141:104347. 10.1016/j.jbi.2023.104347 37030658

[24] Alfattni G Peek N Nenadic G. Extraction of temporal relations from clinical free text: a systematic review of current approaches. J Biomed Inform (2020) 108:103488. 10.1016/j.jbi.2020.103488 32673788

[25] Homburger O Bar K. Large temporal models: unlocking temporal understanding in LLMs for temporal relation classification. In: Proceedings of the 14th International Joint Conference on Natural Language Processing and the 4th Conference of the Asia-Pacific Chapter of the Association for Computational Linguistics (Stroudsburg, PA, USA: The Asian Federation of Natural Language Processing and The Association for Computational Linguistics) (2025), 2156–71. 10.18653/v1/2025.ijcnlp-long.117

[26] Wang J Weiss JC. A large-language model framework for relative timeline extraction from PubMed case reports. AMIA Jt Summits Transl Sci Proc. (2025) 2025:598–606. PMID: 40502242 PMC12150726

